# Binding pocket stabilization by high-throughput screening of yeast display libraries

**DOI:** 10.3389/fmolb.2022.1023131

**Published:** 2022-11-07

**Authors:** Jorge A. Lerma Romero, Christian Meyners, Andreas Christmann, Lisa M. Reinbold, Anna Charalampidou, Felix Hausch, Harald Kolmar

**Affiliations:** ^1^ Institute for Organic Chemistry and Biochemistry, Technical University of Darmstadt, Darmstadt, Germany; ^2^ Centre for Synthetic Biology, Technical University of Darmstadt, Darmstadt, Germany

**Keywords:** protein engineering, transient binding pocket, yeast display, flow cytometry, FKBP, high-throughput screening

## Abstract

Protein dynamics have a great influence on the binding pockets of some therapeutic targets. Flexible protein binding sites can result in transient binding pocket formation which might have a negative impact on drug screening efforts. Here, we describe a protein engineering strategy with FK506-binding protein 51 (FKBP51) as a model protein, which is a promising target for stress-related disorders. High-throughput screening of yeast display libraries of FKBP51 resulted in the identification of variants exhibiting higher affinity binding of conformation-specific FKBP51 selective inhibitors. The gene libraries of a random mutagenesis and site saturation mutagenesis of the FK1 domain of FKBP51 encoding sequence were used to create a yeast surface display library. Fluorescence-activated cell sorting for FKBP51 variants that bind conformation-specific fluorescently labeled ligands with high affinity allowed for the identification of 15 different protein variants with improved binding to either, or both FKBP51-specific ligands used in the screening, with improved affinities up to 34-fold compared to the wild type. These variants will pave the way to a better understanding of the conformational flexibility of the FKBP51 binding pocket and may enable the isolation of new selective ligands that preferably and selectively bind the active site of the protein in its open conformation state.

## Introduction

**FIGURE 1:**
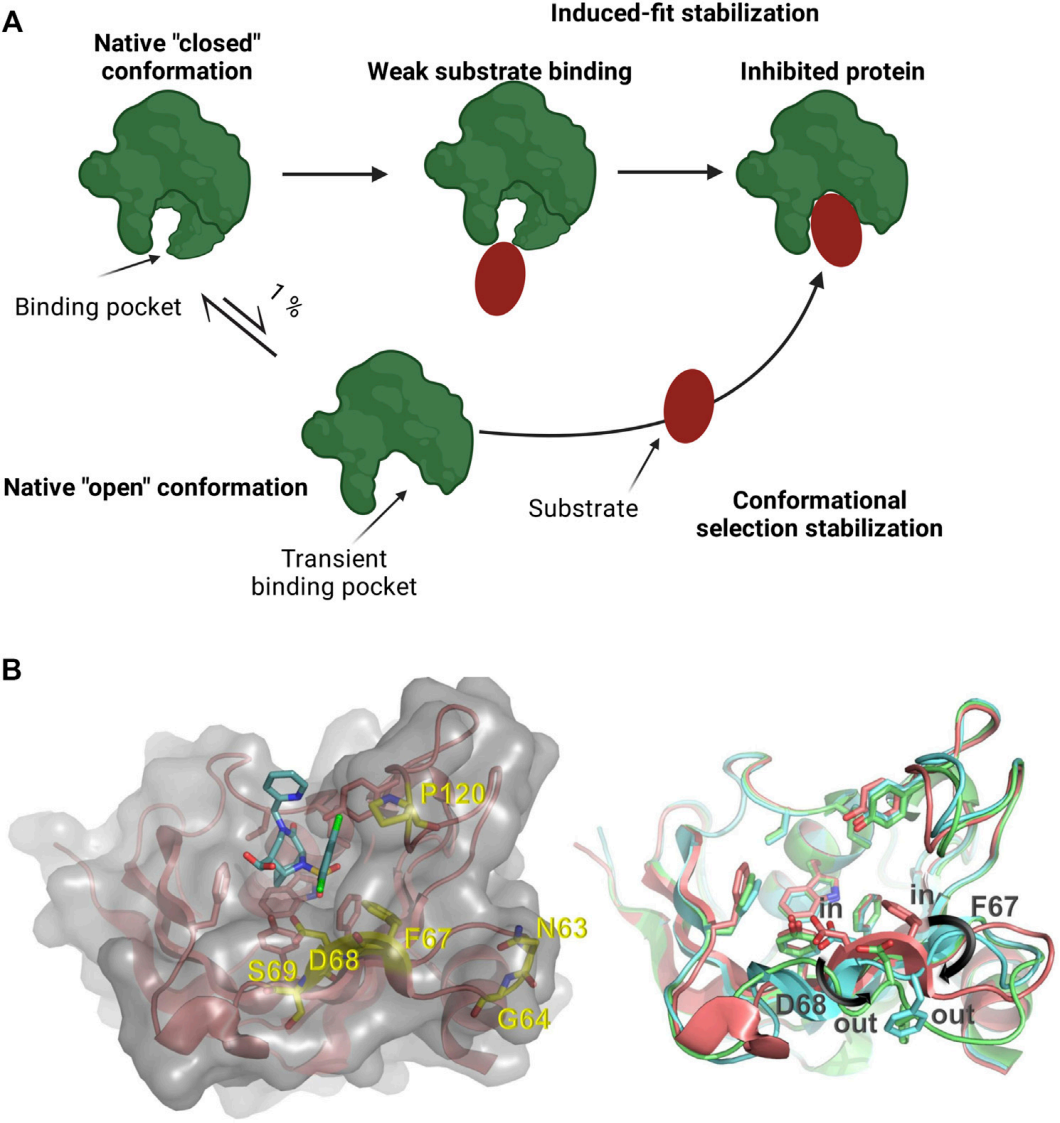
**(A)** Schematic representation of a protein presenting a transient binding pocket stabilized by a substrate *via* induced-fit or conformational selection. Created with BioRender.com **(B)** Left: Structure of the FKBP51 FK1 domain (amino acids: 14–140) bound to FK [431] ligand (**6**) (PDB 5OBK). The FK1 domain is depicted in salmon and important residues of the binding site are shown as sticks. The surface of the FK1 domain is indicated in lighter grey and the ligand is shown as teal sticks. Amino acids identified in the screening are shown as yellow sticks. Right: Overlay of ligand stabilized conformations of FKBP51 FK1. Ligands have been omitted for clarity. Salmon: FKBP51 FK1 domain bound to FK [431] ligand (**6**) (PDB 5OBK) with a F67_in_/D68_in_-conformation; Cyan: FKBP51 FK1 domain bound to iFit1 (PDB 4TW6) with a F67_out_/D68_in_-conformation; Green: FKBP51 FK1 domain bound to macrocyclic ligand (**5**) (PDB 7AWF) with a F67_out_/D68_out_-conformation.

Many proteins, including human drug targets, display large conformational flexibility (Carlson, 2002; Cozzini et al., 2008; Amaral et al., 2017). As a consequence, their binding pockets for small molecule ligands are frequently not well defined and ligand binding can result in conformational changes that eventually lead to the modification or even the appearance of a previously unidentified binding pocket (Monod et al., 1965; Garvey, 2010; Surade & Blundell, 2012; Stank et al., 2016). Upon ligand binding, the opening of a binding pocket displaying a closed conformation can also occur as a result of stabilizing energy contributions of the bound ligand (Figure 1A) (Monod et al., 1965; Garvey, 2010; Voll et al., 2021). Such type of transient binding pockets are difficult to characterize and it is challenging to identify ligands that bind these drug targets with high affinity and selectivity, thereby modifying their function (Zheng et al., 2013; Umezawa & Kii, 2021).

The application of molecular biology methods such as random or focused target protein library generation and high throughput screening for target proteins with altered ligand binding properties can be useful for the identification and characterization of structural and functional consequences of ligand binding to such type of dynamic binding pockets.

A paradigmatic example for a drug target with a transient binding pocket is the FK506 binding protein 51 (FKBP51), encoded by the *FKBP5* gene. FKBP51 is an intracellular protein belonging to the immunophilin family (Sinars et al., 2003; Hähle et al., 2019). Like many other members of the FKBPs family, it has a peptidyl-prolyl isomerase (PPIase) activity, can act as a co-chaperone of heat shock protein 90 (Hsp90) and it plays a role in the regulation of steroid hormone receptor activity (Cioffi et al., 2011; Kolos et al., 2018). In humans, FKBP51 is linked to several stress-related disorders (e.g., anxiety disorders or depression), obesity, type 2 diabetes, and chronic pain. Inhibition of FKBP51 may have beneficial effects on these diseases (Cioffi et al., 2011; Pöhlmann et al., 2018; Häusl et al., 2019).

For a long time, all known FKBP51 ligands such as the immunosuppressive drugs FK506 and rapamycin were unselective, binding with similar affinity to most other members of the immunophilin family with a PPIase domain (Gaali et al., 2015; Kolos et al., 2018). Selective inhibition of FKBP51, however, is thought to be crucial since inhibition of other FKBP members can cause diverse adverse effects. For instance, FKBP12 deficiency caused embryonic lethality due to cardiac defects (Kolos et al., 2018). Furthermore, FKBP52 deletion triggered female infertility and several defects in male sexual development in mice (C. Guy et al., 2015; Kolos et al., 2018; Sivils et al., 2011).

The iFit ligand class (including its analogs SAFit-FL and SAFit1) were the first molecules capable of selectively inhibiting FKBP51 (Gaali et al., 2015). The selectivity of these compounds was enabled by differences in the dynamics in the FK1 domain of this protein family. The binding of these ligands stabilizes a conformational change in the FKBP51 FK1 domain. This conformational change is characterized by the displacement of the F67 side chain (Figure 1B) which creates a transient-binding pocket to accommodate the iFit ligands (Gaali et al., 2015). The required conformational change is energetically highly unfavorable and has to be compensated by additional binding energy of the ligand, which can impose a substantial or even insurmountable barrier for the *de novo* identification of weak ligands, *e.g*., by fragment screening. For FKBP51, the relevant F67_out_-conformation is populated to approximately 0.4% in the apo-state (Jagtap et al., 2019), and various screening approaches did not result in hits for the transient binding pocket. Similarly, the macrocyclic analogs of the iFit ligand class (e.g. Mcyc-TA) require a different rearrangement of the FKBP51 binding pocket (Bracher et al., 2011; Voll et al., 2021). While the displacement of F67 is mainly responsible for the strong selectivity of the iFit ligand against FKBP52, an additional displacement of the D68 residue to an outward conformation improves the macrocyclic ligands selectivity for FKBP51 over FKBP12, FKBP12.6 and FKBP52 (Voll et al., 2021).

Finding a ligand to a target like the transient binding pocket of FKBP51 is a challenging task. Drug design and optimization assisted by a better understanding of the target protein is a logical pathway to obtain a high-affinity ligand to this and many proteins of therapeutic interest. Numerous diseases are caused by the action of effector proteins or any of the subsequent reactions participating in the disease signaling pathway. Normally, the activity of the effector protein is regulated by a small molecule or another protein in the organism (Setiawan et al., 2018). Protein engineering has been an essential tool to elucidate protein structures and determine the protein-drug interactions. The obtained information facilitates the design or discovery of protein inhibitors which may disrupt the action of proteins participating in disease pathways. By optimizing the protein-drug interactions we can improve the binding to the desired target protein and collect information to design an improved ligand in a rapid and iterative manner (Argiriadi et al., 2009; Liang et al., 2021).

It has been shown that by taking advantage of the flexibility or the presence of a transient binding pocket in a target protein, the specificity and selectivity of small molecules might be improved (Kokh et al., 2016; Umezawa & Kii, 2021). In recent years, there had been extensive research to detect transient pockets using *in silico* experiments to design new molecules that can efficiently bind to diverse protein targets (Eyrisch & Helms, 2007; Kokh et al., 2016). Even though the discovery of transient binding pockets is a challenging task and the open conformation state of a protein is an event that happens in less than 1% of the cases in some proteins, the information acquired through these experiments is of great value for the development of new potential therapeutic compounds (Eyrisch & Helms, 2007).

High throughput random mutagenesis and site saturation mutagenesis are powerful protein engineering tools that allow for the identification of amino acids that play an essential role in the structure and function of a protein. Here we describe a protein engineering strategy aimed at enhancing the binding affinity of conformation-specific selective FKBP51 ligands. With the help of fluorescence-activated cell sorting (FACS) of a yeast display library of FKBP51 mutants, a number of variants with improved binding of iFit class ligands were identified and protein crystallization indicated, at least for one variant, that this seems to be due to stabilization of the binding pocket. We expect that these variants will help to identify novel ligand scaffolds that selectively block FKBP51 over other members of the FKBP family. Furthermore, these variants may contribute to a better understanding of the protein-ligand interaction and the dynamics and plasticity of the FKBP51 transient-binding pocket.

## Materials and methods

### Random mutagenesis of FKBP51 (FK1 domain)

The coding sequence of the FK1 domain (1–140) of the FKBP51 (PDB: 3o5e) was used as a template for the generation of genetic diversity of the parent sequence. Sequence diversity was achieved through the introduction of random point mutations. A random mutagenesis reaction was prepared following the protocol of the GeneMorph II random mutagenesis kit (Agilent Technologies). Three different mutation rates were achieved by modifying the template amount (pCT-HsFKBP51 plasmid) in the random mutagenesis reaction. For a mutation frequency of 9–16 mutations/Kbp, 4.5-9 mutations/Kbp and 0–4.5 mutations/Kbp, the required template amount was 50 ng, 250 ng and 900 ng, respectively. The annealing temperature for this reaction was established at 64°C and the pCT_FKBP51_fw and pCT_FKBP51_rv primers (Supplementary Table S1) were used. FKBP51 coding sequence length is 420 bp, therefore the amplification time was 1 min.

### Site-saturation mutagenesis

For the site-saturation mutagenesis, a two-step PCR was performed. In the first PCR step, the degenerated primers (Supplementary Table S1) were paired with the pCT_FKBP51_fw or pCT_FKBP51_rv (e.g., pCT_FKBP51_fw and N63_deg_Rv). Two PCRs for each position were performed, generating two spliced DNA molecules of the FKBP51 gene.

The mutagenesis reactions were performed in a 50 μl volume containing 5X green Quick-Load reaction buffer, ∼20 ng of pCT-HsFKBP51 plasmid as a template, 0.2 μm of each primer, 200 μm of dNTPs, and 1.25 units of OneTaq^®^ Quick-Load^®^ DNA Polymerase (New England Biolabs). Reactions were thermally cycled: 95°C for 2 min, followed by 30 cycles of 95°C for 20 s, 52–56°C for 50 s, and 68°C for 25 s, then a final incubation of 68°C for 5 min. At this step, the mutation was generated in both strands of the DNA sequence of the FKBP51 gene.

To fuse the two fragments, an overlap extension PCR was performed. The purified products of the first PCR step (1 μl each) were mixed with 5X green Quick-Load reaction buffer, 0.2 nM of each primer (pCT_FKBP51_fw and pCT_FKBP51_rv), 200 μm of dNTPs, 1.25 units of OneTaq^®^ Quick-Load^®^ DNA Polymerase (New England Biolabs) and filled up with ddH_2_O to a final volume of 50 μl. Reactions were thermally cycled: 95°C for 2 min, followed by 30 cycles of 95°C for 20 s, 46°C for 50 s, and 68°C for 35 s, then a final incubation of 68°C for 5 min. Reactions were cooled on ice and digested with 5 units of DpnI for at least 1 h at 37°C to cleave methylated parental DNA, but not the newly synthesized mutant DNA molecules. The complete FKBP51 DNA sequence with one codon mutated was then purified and stored at -20°C.

### DNA purification, concentration determination, and sequencing

PCR products and enzymatic restriction reactions were purified by Wizard^®^ SV Gel and PCR Clean-up System Kit from Promega following the manufacturer´s instruction. The purified DNA was recovered in nuclease-free water and the concentration was measured by spectrometry absorbance at 260 nm using the Biospec NanoTM from Shimadsu Europe GmbH. For sequencing, the cleaned-up DNA product was mixed with pCT_seq_up or pCT_seq_lo primer (Supplementary Table S1). The samples were sent for sequencing (SeqLab Göttingen GmbH).

### Yeast library generation

The yeast library was generated *via* homologous recombination. Before the yeast transformation, the destination vector was linearized with the restriction enzymes BamHI (New England Biolabs) and NheI (New England Biolabs). The *Saccharomyces cerevisiae* strain EBY100 [MATa URA3-52 trp1 leu2Δ1 his3Δ200 pep4:HIS3 prb1Δ1.6R can1 GAL (pIU211:URA3)] (Thermo Fisher Scientific) was used for the generation of the FKBP51 mutant library. EBY100 yeast cells were cultivated in Yeast Extract–Peptone–Dextrose (YPD) medium composed of 20 g/L peptone-casein (Carl Roth GmbH &Co.KG), 20 g/L glucose (Carl Roth GmbH &Co.KG), and 10 g/L yeast extract (Sigma-Aldrich).

Electrocompetent yeast cells and libraries were generated following Benatuil *et al.* protocol (Benatuil et al., 2010). Cell transformation was performed using 4 μg digested destination vector (pCT vector) and 12 μg purified PCR product within each transformation reaction. 20 electroporation reactions were performed for the generation of the FKBP51 mutant library in EBY100. The cells were electroporated at 2.5 kV and 25 mF in a 0.2 cm BioRad GenePulser cuvette. The cells were immediately resuspended in a 1:1 mix of 1 M sorbitol: YPD medium and incubated at 30 °C for 1 h. Finally, the cells were collected and cultured in SD-Trp media which contained 20 g/L glucose, 6.7 g/L yeast nitrogen base without amino acids (Becton, Dickinson and Company), 5.4 g/L Na_2_HPO_4_ (Carl Roth GmbH &Co.KG), 8.6 g/L NaH_2_PO_4_.H_2_O (Carl Roth GmbH &Co.KG), and 5 g/L casamino acids. Library sizes were calculated from serial dilution plating of transformed cells.

### FACS screening and sorting

The library cells were grown overnight in SD medium at 30°C and 200 rpm. Afterward, cells were transferred to SG medium (20 g/L galactose, 6.7 g/L yeast nitrogen base without amino acids, 5.4 g/L Na_2_HPO_4_, 8.6 g/L NaH_2_PO_4_.H_2_O, and 5 g/L casamino acids) at 10^7^ cells/ml followed by incubation at 30°C for approximately 24 h. Labeling of cells for FACS analysis or sorting was conducted by washing and resuspending the FKBP51 mutant library with PBS (6.4 mM Na_2_HPO_4_, 2 mM KH_2_PO_4_, 140 mM NaCl, 10 mM KCl) followed by incubation with biotin-conjugated c-Myc antibody (Miltenyi Biotec; diluted 1:75) on ice for approximately 30 min. Afterwards, the cells were washed and resuspended a second time in PBS, followed by staining with secondary labeling reagent Streptavidin conjugated to APC (eBioscience™; diluted 1:75) to differentiate between presenting and non-presenting yeast cells. Besides, 5 nM of SAFit-FL or 20 nM of Mcyc-TA was added to the library sample to sort the protein variants with a high affinity to either of those ligands. All the ligand tracers used for cell sorting had purities of more than 95%. Finally, cells were washed one last time with PBS and resuspended in 1 ml of PBS for FACS analysis. FACS-sorting rounds were either performed on a Sony SH800 cell sorter (Sony) or a BD Influx™ cell sorter. Sorting gate was set to capture approximately 1% of the tracer binding population. For the Sony SH800 cell sorter mOrange fluorochrome configuration (561 nm excitation laser, 583/30 optical filter) was used to measure TAMRA labeled tracers; APC fluorochrome configuration (638 nm excitation laser, 665/30 optical filter) was used to measure APC stained myc-tag. For the BD Influx™ cell sorter a 488 nm excitation laser, 530/40 optical filter was used to measure FITC labeled tracers; 640 nm excitation laser, 670/30 optical filter was used to measure APC stained myc-tag.

The sorted cells were used subsequently for the next sorting round or single clone analysis.

### Colony PCR

A single clone of the *S. cerevisiae* was picked and resuspended in 25 μl of 20 nM NaOH and incubated for 20 min at 98°C. Afterward, a PCR was performed using 2 μl of the yeast cell sample as a template and mixed with 5X green Quick-Load reaction buffer, 0.2 μm of the pCT_seq_up and pCT_seq_lo primer, 200 μm of dNTPs, 1.25 units of OneTaq^®^ Quick-Load^®^ DNA Polymerase (New England Biolabs), and filled up to 50 μL with ddH_2_O. Reactions were thermally cycled: 95°C for 1 min, followed by 30 cycles of 95°C for 20 s, 54°C for 50 s, and 68°C for 45 s, then a final incubation of 68°C for 5 min. The PCR products were analyzed by agarose gel electrophoresis, and if required, sent for sequencing.

### Protein production, purification and characterization

*E. coli* BL21 (DE3) was transformed with each of the FKBP51 variants cloned in pET30b by electroporation at 2.5 kV and 25 mF in a 0.2 cm BioRad GenePulser cuvette. The transformed cells were spread on Double Yeast Tryptone (dYT)-agar plates with kanamycin (0.1% v/v) and were incubated at 37°C overnight. A single colony was picked to start a preculture in dYT medium composed of 16 g/L peptone-casein (Carl Roth GmbH &Co.KG), 10 g/L yeast extract (Sigma-Aldrich), and 5 g/L NaCl with kanamycin (0.1% v/v) and grown overnight at 37°C and 180 rpm. A shaking flask containing 1 L dYT-medium was inoculated to an OD_600_: 0.1, using the overnight culture. The cell culture was incubated at 37°C and 180 rpm until an OD_600_ of 0.6–0.8 was reached. Production was carried out overnight by adding 1 mM isopropyl 1-thio-d-galactopyranoside and incubated the cell culture at 30°C and 180 rpm.

Induced *E. coli* BL21 (DE3) cells containing FKBP51 were precipitated by centrifugation (6,000 rpm, 10 min, 4°C) and lysed by sonication. Cellular debris were removed by centrifugation (13,500 rpm, 15 min, 4°C) and the supernatant was filtered through a 0.45 µm syringe filter.

Utilization of an N-terminal His-tag allowed purification by Ni-NTA affinity chromatography (HisTrap HP - Cytiva). Finally, the recovered fractions were dialyzed against 20 mM HEPES, 150 mM NaCl, pH 8 or PBS pH 7.4. Protein purity was confirmed *via* 10% SDS-PAGE analysis under reducing conditions (Supplementary Figure S7).

In order to purify the FKBP51-G64S variant for crystallization trials, the G64S mutation was introduced into our His-SUMO-FKBP51 (16–140, A19T, C103A, C107I) construct and transformed into *E. coli* BL21 (DE3) cells. A single colony was used to inoculate 50 ml LB medium which was then incubated at 37°C overnight. For the main culture 1 L LB medium was inoculated to an OD600 of 0.1 and incubated at 37°C and 180 rpm until an OD600 of 0.6 was reached. The cell culture was cooled to 25°C, induced by addition of 0.5 mM isopropyl 1-thio-d-galactopyranoside and further incubated for additional 16 h.

The cells were harvested by centrifugation (13,000 × g, 15 min, 4°C) and the cell pellet was solubilized in lysis buffer (20 mM HEPES, 300 mM NaCl, pH 8) supplemented with 1 mM PMSF, 2 mg/ml lysozyme, and 0.1 mg/ml DNase I. After incubation for 1 h, the cells were lysed using sonication and cellular debris were removed by centrifugation (20,000 × g, 30 min, 4°C). The supernatant was loaded on a Nickel-NTA (Machery Nagel) column equilibrated with lysis buffer. The column was washed with 10 column volumes of washing buffer (20 mM HEPES, 300 mM NaCl, 10 mM imidazole pH 8) and the protein was eluted with elution buffer (20 mM HEPES, 300 mM NaCl, 300 mM imidazole pH 8). Target protein containing fractions were dialyzed against 20 mM HEPES, 150 mM NaCl, pH 8 and the His-SUMO tag was cleaved by addition of recombinant Ulp1. The cleaved His-SUMO tag was removed by passing the protein mixture through a Nickel-NTA column. The FKBP51-G64S containing flow-through was finally purified by size exclusion chromatography using a HiLoad^®^ 16/600 Superdex^®^ 75 pg column (Cytiva) equilibrated with 20 mM HEPES, 20 mM NaCl, pH 8. The pure protein was concentrated to 20 mg/ml using an Amicon^®^ Ultra 2 ml centrifugal filter, flash frozen in liquid nitrogen, and stored at −80°C until used further.

### Affinity measurement by fluorescence polarization

All ligands and tracers used for fluorescence polarization assays had purities of more than 95%. The following ligands and tracers were used for fluorescence polarization and FACS screening experiments:

•SAFit-FL tracer (**1**): Fluorescein conjugated analog of the iFit ligand class.
•Mcyc-TA tracer (**2**): TAMRA conjugated macrocyclic ligand (**5**).
•FK [431]-TA tracer (**3**): TAMRA conjugated FK [431] ligand (**6**).
•SAFit1 ligand (**4**): analog of the iFit ligand class.
•macrocyclic ligand (**5**):macrocyclic analog of the SAFit class ligands.
•FK [431] ligand (**6**):bicyclic analog of the immunosuppressive drug FK506

The binding of the generated FKBP51 variants to different conformation-sensitive FKBP ligands was investigated by fluorescence polarization assays. Therefore, a serial dilution of the respective FKBP51 variant in assay buffer (20 mM HEPES pH 8.0, 150 mM NaCl, 0.015% Triton X-100) was placed in a 384-well assay plate and a defined amount of the respective fluorescent tracer (0.5 nM of the SAFit based tracer SAFit-FL (**1**), 5 nM of the macrocyclic tracer Mcyc-TA **2**) or 1 nM of the FK [431]-TA **3**) in assay buffer was added to the protein buffer mixture. After incubation for 30 min at room temperature, the fluorescence polarization was measured with a plate reader. The obtained results for each 3 independent experiments were normalized with respect to the maximal binding signal and fitted to a one-site binding model as described by Wang et al., 1992 yielding the respective binding constants.

$$tracer\;bound=\frac{100}{L_{t}}\times 0.5\times \left(R_{t}+L_{t}+K_{D}-\sqrt{{\left(R_{t}+L_{t}+K_{D}\right)}^{2}-4\times L_{t}R_{t}}\right)$$

With *L*_*t*_, total concentration of the tracer, *R*_*t*_, total concentration of the receptor and *K*_*D*_ binding constant of the complex RL.

In order to rule out artifacts introduced by the fluorophore of the tracers, competitive fluorescence polarization assays were carried out. Therefore, a serial dilution of an FK [431] ligand (**6**), SAFit1 (**4**), or a macrocyclic ligand **5**) (Supplementary Figure S8) in assay buffer was placed in a 384-well assay plate. To the compounds, a mixture of the respective protein (20 nM WT, 10–40 nM G64S or 80–100 nM D68Y) and 1 nM of the FK [431]-TA in assay buffer was added. After incubating for 30 min at room temperature, the fluorescence polarization was measured. The obtained results for each 3 independent experiments were normalized with respect to the maximal binding and fitted to a competitive binding model as described by Wang, 1995 yielding the respective binding constants.

$$tracer\;bound=100\times \frac{\left\{2\times \sqrt{\left(a^{2}-3b\right)}\times \cos\left(\theta /3\right)-a\right\}}{3\times K_{D}+\left\{2\sqrt{\left(a^{2}-3b\right)}\times \cos\left(\theta /3\right)-a\right\}}$$

$$a=K_{D}+K_{I}+L_{t}+I_{t}-R_{t}$$

$$b=K_{I}\left(L_{t}-R_{t}\right)+K_{D}\left(I_{t}-R_{t}\right)+K_{D}K_{I}$$

$$c=-K_{D}K_{I}R_{t}$$

$$\theta =arc\cos\frac{-2a^{3}+9ab-27c}{2\sqrt{{\left(a^{2}-3b\right)}^{3}}}$$

With *L*_*t*_, total concentration of the tracer, *R*_*t*_, total concentration of the receptor, *K*_*D*_, binding constant of the complex RL, *I*_*t*_, total concentration of the titrated ligand and *K*_*I*_, binding constant of the complex RI.

**Protein crystallization**

For the crystallization of the FKBP51-G64S complexes, each complex was prepared by mixing FKBP51FK1 A19T, G64S, C103A, C107I (14–140) at 15 mg/ml with a slight molar excess of SAFit1 (**4**), macrocyclic ligand **5**) or FK [431] ligand (**6**), previously dissolved at 20 mM in DMSO. Crystallization was performed at room temperature using the hanging drop vapour-diffusion method by equilibrating mixtures of 1 µL protein complex and 1 µL reservoir against 500 µL reservoir solution containing 12% (**4**), 30% **5**) or 40% **6**) PEG-3350, 0.2 M NH_4_-acetate, and 0.1 M HEPES-NaOH pH 7.5. The crystals were fished, cryoprotected with 30% PEG-3350, 20% glycerol, 0.2 M NH_4_-acetate, and 0.1 M HEPES-NaOH pH 7.5 and flash frozen in liquid nitrogen.

The crystallographic experiments were performed on the BL14.1 beamline at the Helmholtz-Zentrum BESSY II synchrotron, Berlin, Germany (Gerlach et al., 2016). Diffraction data were integrated with XDS implemented in XDSapp3 and further processed with the implemented programs of the CCP4i and CCP4i2 interface (Collaborative Computational Project, N. 4, 1994; Kabsch, 2010; E. Potterton et al., 2003; L. Potterton et al., 2018; Sparta et al., 2016; Winn et al., 2011). The data reduction was conducted with Aimless (Evans, 2011; Evans & Murshudov, 2013). The crystal structure was solved by molecular replacement using Phaser. Iterative model improvement and refinement were performed with Coot and Refmac5 (Murshudov et al., 1997, 2011; Vagin et al., 2004; McCoy et al., 2007; Emsley et al., 2010; Winn et al., 2011; Nicholls et al., 2012). The dictionaries for the compounds were generated with PRODRG implemented in CCP4i (van Aalten et al., 1996). Residues facing solvent channels without detectable side chain density were truncated.

## Results

**FIGURE 2:**
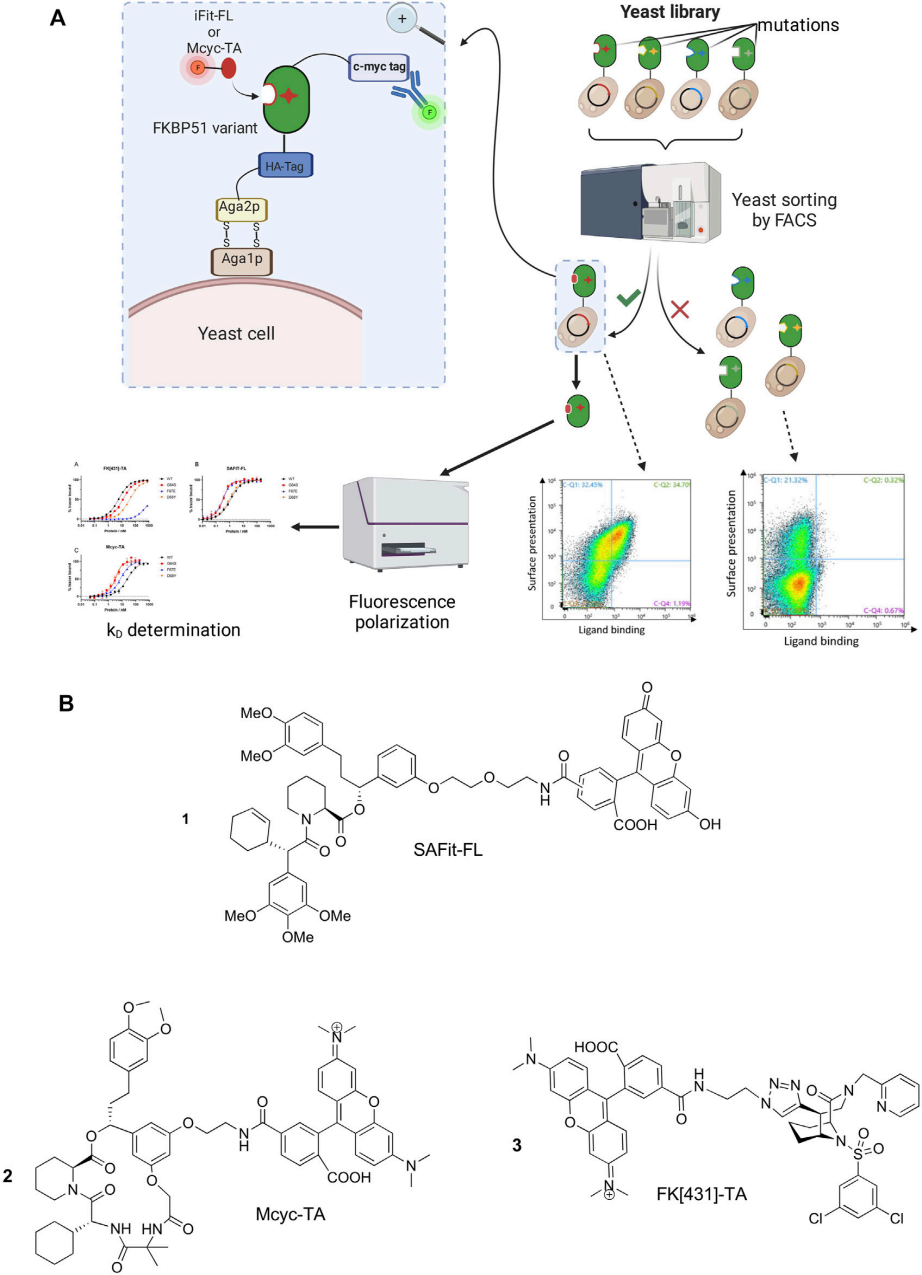
**(A)** Schematic representation of the yeast surface display (YSD) presenting mutated FKBP51 variants, FACS-based screening strategy and affinity determination. Created with BioRender.com. **(B)** Chemical structure of the FKBP tracers SAFit-FL _(out/in)_ (1), Mcyc-TA _(out/out)_ (2), and FK [431]-TA _(in/in)_ (3).

In order to identify FKBP51 variants with improved binding affinities to selective FKBP51 ligands we aimed to combine protein engineering strategies with conformation-specific ligands for the selection rounds. Therefore, we started by synthesizing a pool of randomly mutated FKBP51 DNA sequences covering the whole FK1 domain and used them to generate a yeast display library with a size of approximately 3.5*10^6^ clones. The library was screened for three rounds *via* FACS (Figure 2A) using the two known conformation-specific FKBP51 tracers SAFit-FL **1**) and Mcyc-TA (**2**). For the characterization FK [431]-TA **3**) was included as a third tracer (Figure 2B). SAFit-FL **1**) is a fluorescent analog of the iFit ligand class, which binds preferentially to the F67^out^/D68^in^-conformation of FKBP51 (Gaali et al., 2015). Mcyc-TA _(out/out)_ (**2**, compound 14 in (Voll et al., 2021)) is a macrocyclic analog of the SAFit class ligands that binds to an F67^out^/D68^out^ conformation and, unlike the previous generations of iFit ligands, displays additional selectivity over FKBP12 and FKBP12.6 (Voll et al., 2021). The fluorescently labeled FK [431]-TA _(in/in)_ **3**) is a bicyclic analog of the FK506, which binds to the canonical F67^in^/D68^in^-conformation. In the following the preferred binding modes of these ligands are abbreviated with *out/in*, *out/out*, and *in/in*, respectively.

**FIGURE 3:**
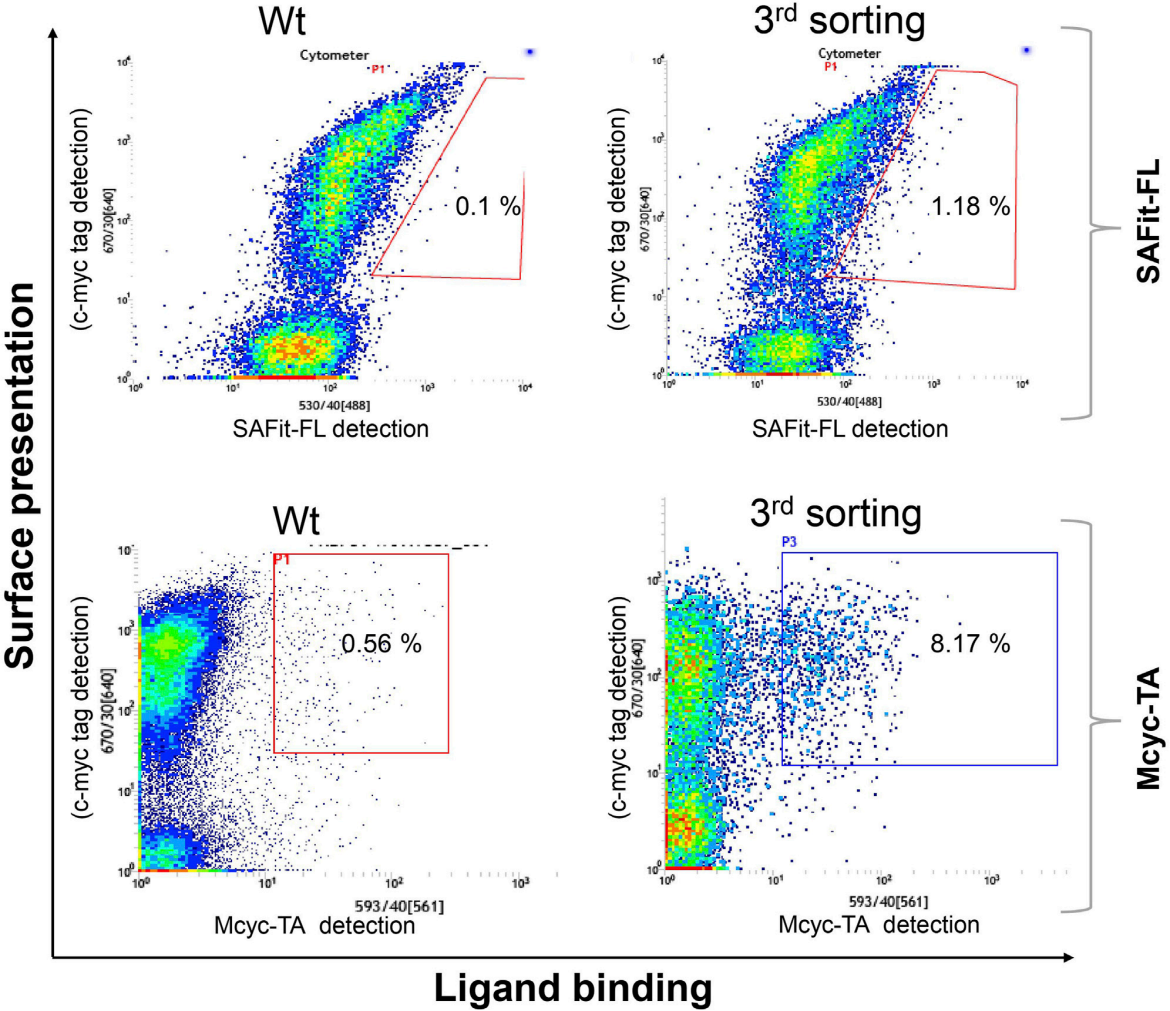
Random mutagenesis YSD library screening and sorting. Cells showing both surface presentation (c-myc tag detection) and ligand binding signal (SAFit-FL _(out/in)_ or Mcyc-TA _(out/out)_) were sorted to enrich the population of ligand binder variants after random mutagenesis of the FK1 coding sequence. Sorting gate was set to capture approximately 1% of the tracer binding population and used for subsequent sorting rounds or single clone analysis.

Two library screening campaigns with three sorting rounds each using 5 nM of SAFit-FL_(out/in)_ **1** or 20 nM of Mcyc-TA _(out/out)_ **2**, respectively, revealed accumulation of FKBP51 variants with enhanced ligand binding compared to wildtype protein (Figure 3). From a gene sequencing of 20 individual clones from our third round FACS sorting of the FKBP51 random mutagenesis library (Supplementary Figure S1-S5), in total seven different protein variants were obtained: G64E, G64S, F67S, D68N, D68Y, S69Y, and P120R.

Interestingly, six out of the seven protein variants displayed an amino acid exchange in the region spanning G64 to S69 (Supplementary Figure S9). It has been shown that the higher conformational plasticity of the FKBP51 *β*_3_ strand and the *β*_4-5_ interconnecting loop (Y113-T127) differs in a great manner to FKBP52 (Hähle et al., 2019). These differences in the conformational plasticity of FKBP51 allow F67 to be displaced to an out-conformation creating a transient binding pocket in the protein (Gaali et al., 2015; Hähle et al., 2019). Aimed at increasing the number of possible mutants and to find variants with further improved FKBP51 ligand interaction, we generated a variant subset by site saturation mutagenesis for positions 63 to 70. By mutating a single amino acid position at a time with degenerate primers (NNK) coding for all 20 amino acids for the 8 selected residues, we expect a combined library consisting of 160 variants of the FKBP51 FK1 domain.

**FIGURE 4:**
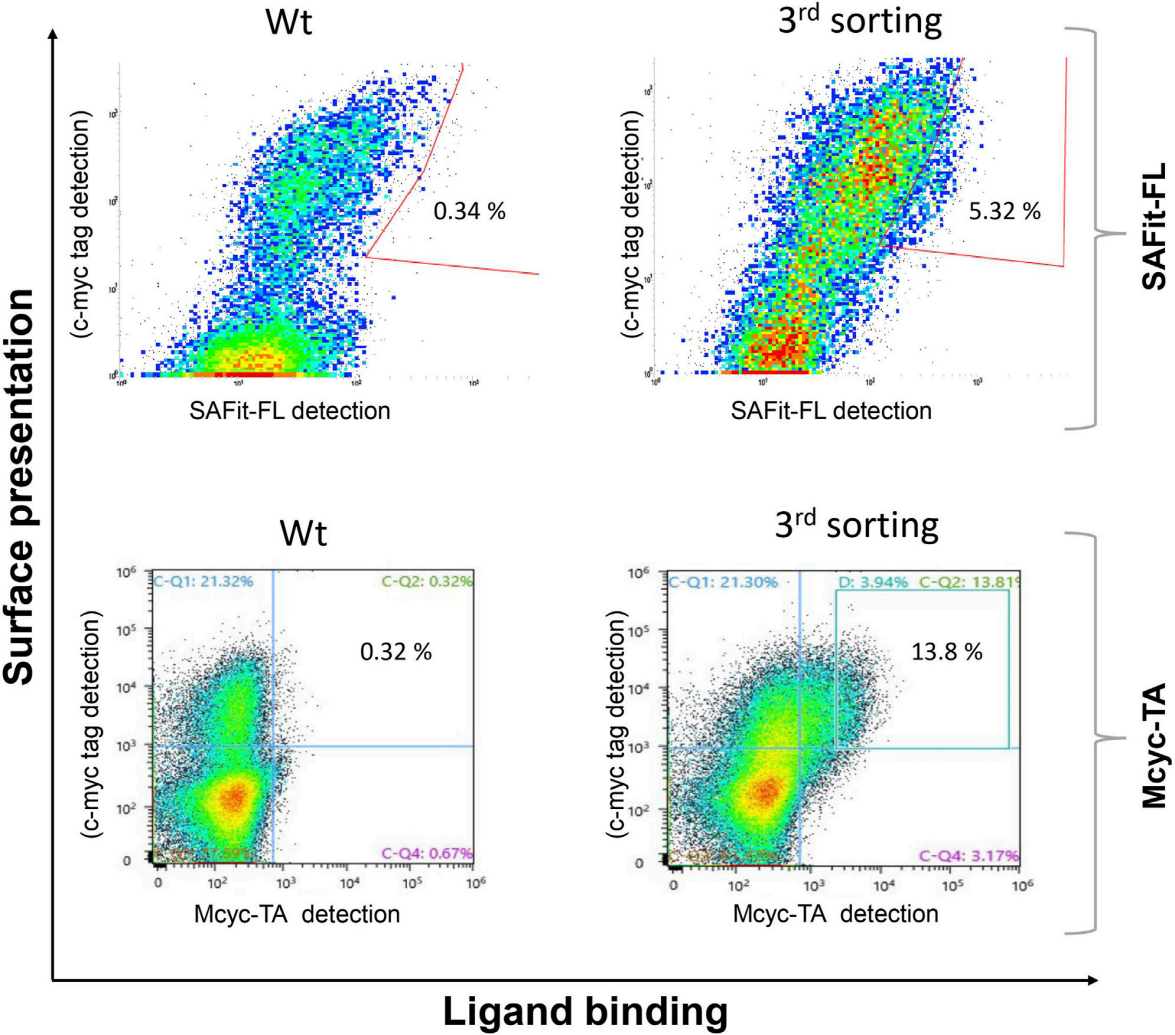
Site saturation mutagenesis YSD library screening and sorting. Cells showing both surface presentation (c-myc tag detection) and ligand binding signal (SAFit-FL _(out/in)_ or Mcyc-TA _(out/out)_) were sorted to enrich the population of ligand binder variants after site saturation mutagenesis for positions 63 to 70 of the FKBP51 sequence. Sorting gate was set to capture approximately 1% of the tracer binding population and used for subsequent sorting rounds or single clone analysis.

The combined Site Saturation Mutagenesis (SSM) PCR products were used to create a yeast library. After three sorting rounds *via* FACS (Figure 4), 13 different FKBP51 variants were identified containing amino acid exchanges in five of the chosen eight positions (Table 1 and Supplementary Figures S1–S5). The variants G64A, G64S, and F67W were found after sorting the SSM library with both ligands, independently. The variant D68Y, which also was found during the random mutagenesis library sorting was found in 16 of the 20 picked yeast colonies sorted with the Mcyc-TA ligand _(out/out)_ **2**_,_ indicating a strong enrichment.

**TABLE 1:** Identified FKBP51 variants after FACS sorting of the random mutagenesis and SSM yeast library with SAFit-FL _(out/in)_ or Mcyc-TA tracers.

FKBP51 variant	Sorted with		Library source	
	SAFit-FL _(out/in)_	Mcyc-TA _(out/out)_	Random mutagenesis	SSM
N63A	✓			✓
N63G	✓			✓
G64A	✓	✓		✓ [3 SAFit-FL]
G64D	✓			✓
G64E	✓	✓	✓	✓
G64R	✓	✓	✓	✓
G64S	✓	✓		✓ [3 SAFit-FL]
G64K	✓			✓
G64T	✓			✓
F67E	✓			✓
F67R	✓			✓
F67S	✓		✓	
F67W	✓	✓		✓ [3 SAFit-FL]
D68N	✓		✓	
D68Y		✓	✓	✓ [16 Mcyc-TA]
S69Y	✓		✓	
P120R	✓		✓	

The number of times that a mutation was found out of 20 picked colonies is depicted in brackets.

**FIGURE 5:**
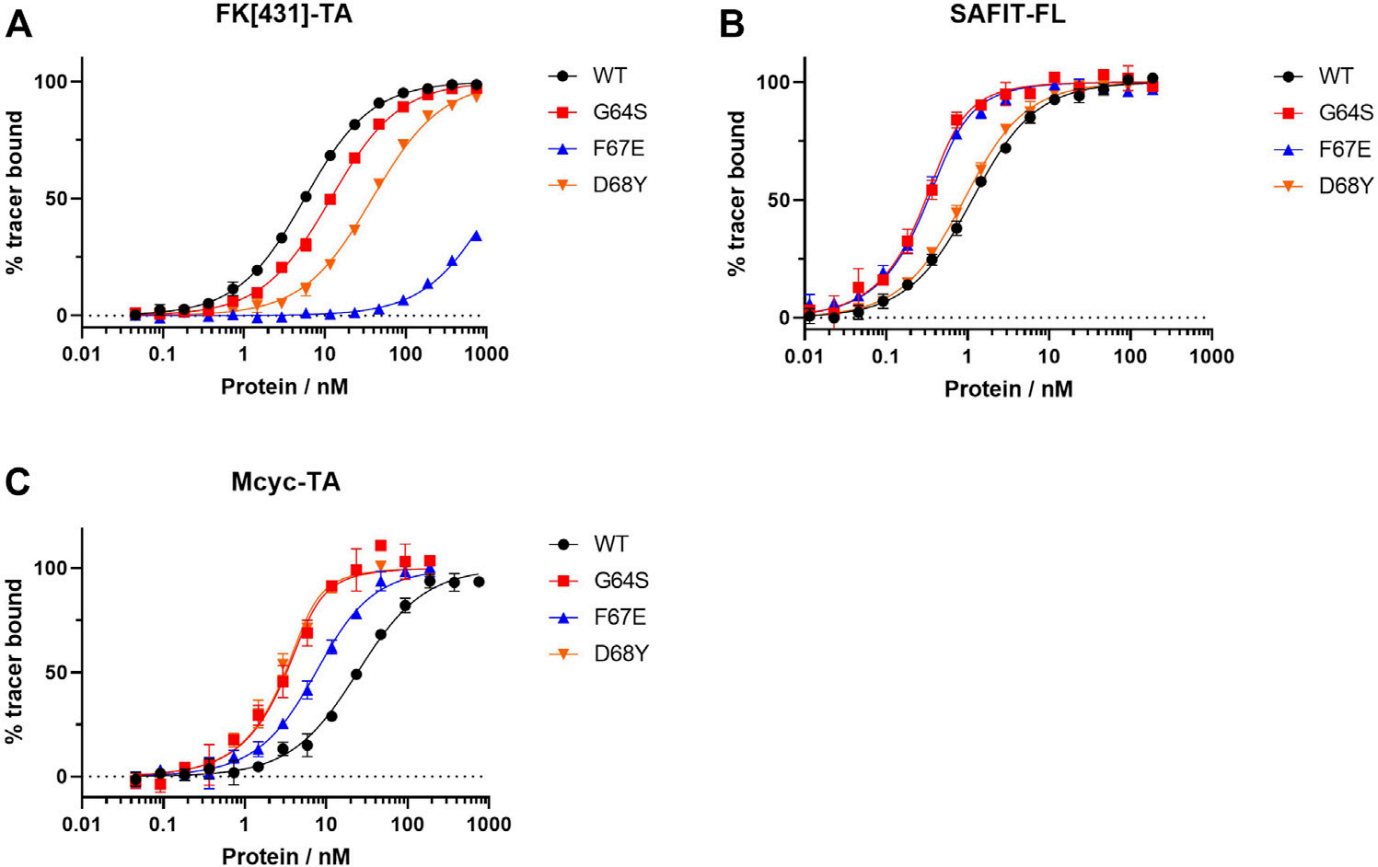
Fluorescence polarization assay for the best three FKBP51 variants using **(A)** the canonical FK1 tracer FK [431]-TA _(in/in)_, **(B)** SAFit-FL _(out/in)_ and **(C)** Mcyc-TA _(out/out)_.

All identified variants were expressed in *E. coli BL21(DE3)* and purified by immobilized metal ion affinity chromatography (Supplementary Figure S7). To quantitatively assess the contribution of each residue replacement to ligand binding the affinities for binding ligands **1, 2** and **3** were determined *via* measurement of concentration-dependent change of fluorescence polarization (Table 2 and Figure 5). Additionally, competitive fluorescence polarization assays were carried out to validate that the binding results were not influenced by the fluorophore of each ligand (Supplementary Table S2, Supplementary Figure S6). An analysis of the obtained data revealed that most variants bound at least one of the ligands **1** or **2** with enhanced affinities compared to wild-type FKBP51.

**TABLE 2:** Ligand binding affinities measured by fluorescence polarization and fold change in Kd improvement with the canonical FK [431]-TA _(in/in)_ and the two FKB51 specific tracers (SAFit-FL _(out/in)_ and Mcyc-TA _(out/out)_).

FKBP51 variant	Tracer/K_d_-value [nM]			Fold change in K_d_ improvement		
	FK [431]-TA (in/in)	SAFit-FL (out/in)	Mcyc-TA (out/out)	FK [431]-TA (in/in)	SAFit-FL (out/in)	Mcyc-TA (out/out)
WT	5.1 ± 0.1	0.92 ± 0.05	24 ± 2	1	1.00	1.00
N63A	6.0 ± 0.2	0.36 ± 0.02	4.3 ± 0.5	0.850	2.56	5.58
N63G	6.0 ± 0.2	0.33 ± 0.02	7.6 ± 0.9	0.850	2.79	3.16
G64A	61 ± 2	0.73 ± 0.05	4.2 ± 0.5	0.084	1.26	5.71
G64D	17 ± 1	0.50 ± 0.04	8.7 ± 1	0.300	1.84	2.76
G64E	19 ± 1	0.40 ± 0.03	3.3 ± 0.3	0.268	2.30	7.27
G64R	32 ± 2	1.1 ± 0.1	9.9 ± 1	0.159	0.84	2.42
G64S	11 ± 1	0.09 ± 0.01	0.7 ± 0.2	0.464	10.22	34.29
G64K	18 ± 1	0.93 ± 0.04	3.5 ± 0.5	0.283	0.99	6.86
G64T	61 ± 2	1.2 ± 0.1	17 ± 1	0.084	0.77	1.41
F67E	1331 ± 66	0.11 ± 0.01	5.0 ± 0.5	0.004	8.36	4.80
F67R	312 ± 14	2.3 ± 0.2	24 ± 3	0.016	0.40	1.00
F67S	1245 ± 60	0.68 ± 0.05	21 ± 2	0.004	1.35	1.14
F67W	1331 ± 66	1.6 ± 0.1	16 ± 1	0.004	0.58	1.50
D68N	146 ± 6	1.8 ± 0.1	6 ± 1	0.035	0.51	4.00
D68Y	38 ± 2	0.69 ± 0.03	0.7 ± 0.1	0.134	1.33	34.29
S69Y	66 ± 2	0.93 ± 0.05	17 ± 1	0.077	0.99	1.41

While most variants exhibited only moderately improved affinities, three variants stood out with 8- to 34- fold increased binding affinities. The FKBP51 variant G64S had a remarkable improvement in the binding affinity of both FKBP51-selective ligands (Figure 5). With a K_d_ of 0.09 ± 0.01 nM for SAFit-FL _(out/in)_ and 0.7 ± 0.2 nM for Mcyc-TA _(out/out)_, the affinity of this variant showed a 10- and 34-fold increase, respectively, while no improvement of ligand binding was seen for the canonical inhibitor FK [431]-TA _(in/in)_ **3**. Likewise, the D68Y variant was another of the mutations that presented a remarkable improvement on the binding of Mcyc-TA _(out/out)_ with a 34-fold increase compared to wildtype FKBP51, whereby no improvement for binding of **1** or **3** was observed. The third interesting variant is the F67E with an 8-fold tighter binding to SAFit-FL _(out/in)_ compared to wildtype FKBP51 and a moderately improved binding for Mcyc-TA _(out/out)_. Interestingly, none of the variants indicated improved binding for the FK [431]-TA _(in/in)_ and in fact, for most variants, a decrease in the binding affinity could be observed. This effect was especially pronounced for all variants with a substitution at position F67, which displayed a dramatic decrease in the binding affinity (Figure 5A).

**FIGURE 6:**
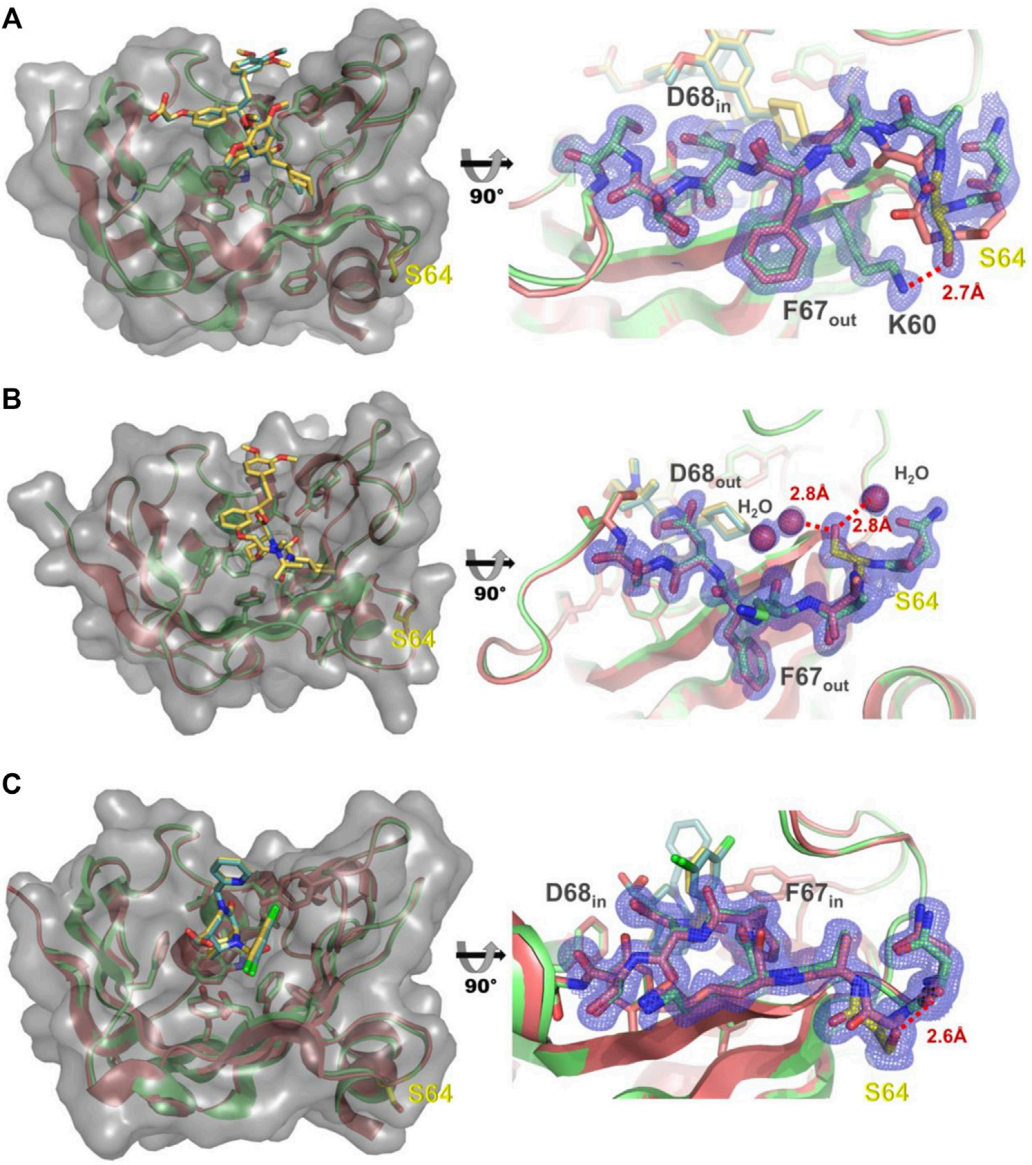
**(A)** Left: Structure of FKBP51-G64S (green cartoon, PDB: 7R0L) bound to SAFit1 (golden sticks) superposed with the FKBP51:iFit1 complex (cyan cartoon, teal sticks, PDB: 4TW6). The surface of FKBP-G64S is indicated in lighter grey. Right: Detailed view of the glycine to serine substitution. The observed electron density contoured at 1σ for residues 60 and 63–70 is shown as blue mesh. The hydrogen bond between lysine 60 and serine 64 is depicted as red line. **(B)** Left: Structure of FKBP51-G64S (green cartoon, PDB: 8BA6) bound to macrocyclic ligand (**5**) (golden sticks) superposed with the FKBP51 bound to the same ligand (cyan cartoon, teal sticks, PDB: 7AWF). The surface of FKBP-G64S is indicated in lighter grey. Right: Detailed view of the glycine to serine substitution. The observed electron density contoured at 1σ for residues 63–69 is shown as blue mesh. The hydrogen bonds between serine 64 and two water molecules are depicted as red lines. **(C)** Left: Structure of FKBP51-G64S (green cartoon, PDB: 8BAJ) bound to FK [431] ligand (**6**) (golden sticks) superposed with the FKBP51 bound to the same ligand (cyan cartoon, teal sticks, PDB: 5OBK). The surface of FKBP-G64S is indicated in lighter grey. Right: Detailed view of the glycine to serine substitution. The observed electron density contoured at 1σ for residues 63–69 is shown as blue mesh. The hydrogen bond between asparagine 63 and serine 64 is depicted as red line.

As the G64S variant showed the strongest improvements in the binding affinity of both FKBP51-selective ligands and its role in the formation of the transient binding pocket is not obvious, we decided to explore the molecular basis for the binding affinity enhancement in more detail. Therefore, we solved the crystal structures of FKBP51-G64S in complex with SAFit1 _(out/in)_ (PDB: 7R0L), macrocyclic ligand **5**) _(out/out)_ (PDB: 8BA6) and FK [431] ligand **6**) _(in/in)_ (PDB: 8BAJ, data collection and refinement statistics in Supplementary Table S4). Overall, the complexes crystallized in very similar conformations as observed for wild-type FKBP51 in complex with the respective ligands (PDB: 4TW6, 7AWF, 5OBK Figure 6). A structural alignment of the respective structure pairs indicates RMSD values > 1 Å only for FKBP51-G64S:SAFit1 _(out/in)_ amino acids 62–65 and for FKBP51-G64S:FK [431] ligand **6**) _(in/in)_ G43 (Supplementary Figure S11). Interestingly, for none of the three ligands a direct interaction between the ligand and the newly introduced serine 64 can be observed. In the FKBP51-G64S:SAFit1 _(out/in)_ complex the loop involving serine 64 is slightly shifted and S64 engages in a hydrogen bond with K60 (Figure 6A). In complex with the macrocyclic ligand **5**) _(out/out)_ S64 is not shifted in comparison to wild-type FKBP51 G64 and engages in a hydrogen bond network with water molecules (Figure 6B). In the FKBP51-G64S: FK [431] ligand **6**) _(in/in)_ complex only S64 is slightly shifted and comes in close contact to the carbonyl oxygen of N73 (Figure 6C).

## Discussion

The FK506-binding protein 51 (FKBP51) has been identified as a key player in several diseases such as chronic pain, obesity, and like stress-related disorders (Cioffi et al., 2011; Pöhlmann et al., 2018; Häusl et al., 2019). A linear analog of FK506 called SAFit was shown to be highly selective for FKBP51 over its closest homologue FKBP52 (Gaali et al., 2015). It has been shown that the displacement of phenylalanine 67 from the binding site to an outward position is the key observation during the binding of SAFit-like and also of Mcyc-TA-like ligands and is responsible for the observed selectivity of these ligand classes. In this study, we performed random mutagenesis over the coding sequence for the FKBP51 FK1 domain and applied a high throughput yeast display screening strategy to identify variants with enhanced affinity to fluorescently labelled conformation-specific ligands. Not unexpectedly, the phenylalanine 67 amino acid position was also identified in our HTS screen as a key residue for selective SAFit _(out/in)_ and Mcyc-TA _(out/out)_ ligand binding. The substitution for glutamic acid resulted in a substantial improvement of SAFit-FL _(out/in)_ and Mcyc-TA _(out/out)_ binding. If a similar displacement for E67 is assumed as it is observed for F67, E67 would locate between K58 and K60, whose positive charges may stabilize E67 in the outward conformation. To further corroborate the importance of the phenylalanine 67 to the specific ligand binding we observed in our results that all variants with a mutation at position F67, presented a drop in the binding affinity to FK [431]-TA _(in/in)_. These mutations hamper the binding to FK [431]-TA _(in/in)_, which binds to the F67^in^/D68^in^-conformation of the protein. Analogous binding experiments of the FK [431] ligand _(in/in)_ to F67V and F67Y variants revealed opposing results (Jagtap et al., 2019). While F67Y displayed a decrease in its binding affinity (similar to all our F67 variants), F67V had a slight improvement of the Kd value compared to the WT.

Similar to phenylalanine 67, the displacement of D68 from the binding pocket is a hallmark of the binding of Mcyc-TA-like ligands (but not of SAFit-like ligands). Upon binding of Mcyc-TA _(out/out)_ to wildtype FKBP51, D68 is displaced by the ligand, which takes its place as a hydrogen bond acceptor for the Y57 hydroxyl group (Voll et al., 2021). Lowering the energy needed for this conformational rearrangement would likely result in an increased binding affinity of Mcyc-TA _(out/out)_ and this might be indeed the case for the improved binding properties of the D68N variant. However, the improvement of the binding affinity of Mcyc-TA _(out/out)_ to the D68Y and the fact that no other amino acids substitutions were observed in this position suggests a more complex explanation for D68Y. A tyrosine in position 68 cannot easily exist in the canonical F67^in^/Y68^in^-conformation as observed for the apo state (F67^in^/D68^in^) of wildtype FKBP51 due to some steric clashes (Supplementary Figure S10) (Bracher et al., 2011). We postulate that in addition to destabilizing the F67^in^ conformation, the phenol side chain of Y68 is especially well suited to stabilize a F67^out^/Y68^out^ conformation.

In contrast to F67 and D68, the contribution of glycine 64 to the stabilization of the binding pocket is less obvious. However, 7 out of 17 protein variants that were found in these experiments displayed a mutated G64 suggesting an important role of G64 for the binding of FKBP ligands. The role of glycine in proteins is unique as it lacks a sidechain which allows glycine to adopt unique backbone conformations. Indeed, G64 consistently adopts ϕ/ψ angles of approx. 91°/-9°, respectively, in the available FKBP51 apo structures or cocrystal structures with canonical ligands (*e.g*. 3O5Q, 3O5R, 5OBK, 7APT, 7APW) (Bischoff et al., 2014; Gopalakrishnan et al., 2012; Kolos et al., 2021; Pomplun et al., 2018; Y. Wang et al., 2013), thus populating a conformation allowed for glycine but disfavored for other amino acids. Moreover, G64 consistently adopts conformations of ϕ/ψ angles of approx. 68°/25° for SAFit-like cocrystal structures (F67_out_/D68_in_) (Feng et al., 2015, 2020; Gaali et al., 2015, 2016; Bauder et al., 2021) and of approx. -74°/149° for Mcyc-TA-like cocrystal structures (F67_out_/D68_out_) (Supplementary Table S3) (Voll et al., 2021). The conformation of G64 thus seems to be coupled to the conformation of the β3a strand, where the canonical F67_in_/D68_in_ conformation favors a glycine-specific conformation at position 64, whereas F67_out_/D68_in_ or F67_out_/D68_out_ do not. A similar observation can be made for the FKBP51-G64S structures. Here, serine 64 adopts ϕ/ψ angles of 53.5°/26° in complex with SAFit1 _(out/in)_ and -77°/149.8° in complex with macrocyclic ligand **5**) _(out/out)_. Interestingly, in the complex with FK [431] ligand **6**) _(in/in)_ S64 adopts with observed ϕ/ψ angles of 89.4°/-8.2° a high energy conformation similar to G64 highlighting the importance of this conformation for the binding of canonical FK [431] _(in/in)_ ligands. In the case of serine 64 this conformation seems to be tolerated by establishing a hydrogen bond to the carbonyl oxygen of N73 upon binding of FK [431] ligand **6**) _(in/in)_ (Figure 6C). This seems not to be case for the other G64 variants as these show a more pronounced decrease in binding affinity for FK [431]-TA _(in/in)_ especially observable for G64A and G64T.

The allosteric destabilization of the F67 in-state by requiring a high energy backbone conformation on position 64 is certainly not sufficient to explain the unique improvement of binding affinities observed for the G64S variant. For the binding of SAFit-FL _(out/in)_ only a serine substitution on position 64 shows a strong improvement in binding affinity. Strikingly, in the SAFit1-bound state S64 forms a hydrogen bond with the side chain of lysine 60 (Figure 6A), which seems not to be possible for the other G64 variants. In contrast to SAFit-FL _(out/in)_ several G64 variants show improved binding constants for Mcyc-TA _(out/out)_ but again a serine substitution was strongly preferred. The structure of the FKBP51-G64S: macrocyclic ligand **5**) _(out/out)_ complex reveals that serine 64 participates in a water mediated hydrogen bond cluster stabilizing the conformation of the residues 62–66 (Figure 6B). From the determined affinity data, it seems that besides G64S, only G64K and G64E are to some extend able to integrate reasonably well into this water cluster. Taken together, our results for the FKBP51-G64 variants strongly suggest that the improvement of the binding affinity of our conformation-specific ligands is due to a combination of destabilization of unproductive protein conformations, augmented for very favorable cases by specific stabilization of the productive conformation, and not due to novel contacts with the ligand.

The P120R is the only residue replacement outside the residue 63 to 69 amino acid stretch that we have identified in this work to enhance the binding of ligand **1** in FACS measurements. It has been reported that the carbonyl oxygen atom of P120 in FKBP51 is directed toward the binding pocket and its *cis* conformation differs from the trans conformation adopted in FKBP52. Moreover, it has been suggested that targeting the amino acids between positions 119 and 124 (L/P119 loop) might have an effect on steroid hormone receptors modulation (Schmidt et al., 2012). Additional structural and functional analysis of the influence of these residues on SAFit-FL _(out/in)_ and Mcyc-TA _(out/out)_ ligand binding will be required to understand, whether this region plays a role in the stabilization of the binding pocket of FKBP51.

In conclusion, we established a combined yeast display and FACS sorting strategy to identify variants of the FKBP51 FK1 domain with improved binding properties for conformation-specific tracers of FKBP51. Most of the 17 identified variants displayed an improved binding to either or both of the FKBP51 specific ligands (SAFit-FL _(out/in)_ and Mcyc-TA _(out/out)_). Of all the found variants, G64S, D68Y, and F67E mutations presented the most significant K_d_ improvement. These three FKBP51 variants will be further investigated in the future to elucidate if they can be used for the identification of new ligand scaffolds targeting the transient binding pocket of FKBP51. Furthermore, we hope to obtain further insights how these mutations affect the protein dynamics and the molecular details of transient pocket formation and ligand recognition. Collectively, our results show how protein engineering using yeast display and conformation-specific tracers can be used to identify variants with improved binding affinities most likely by stabilizing the binding pocket of a protein. As soon as conformation-specific tracers are available, this approach may facilitate drug discovery by substituting target proteins with inaccessible binding pockets with the improved variants for ligand screening.

## Data Availability

The original contributions presented in the study are included in the article/Supplementary Material, further inquiries can be directed to the corresponding authors.
