# Supplementary material for: Binding pocket stabilization by high-throughput screening of yeast display libraries
Source: Front Mol Biosci. 2022 Nov 7;9:1023131. doi: 10.3389/fmolb.2022.1023131 (PMC9676650; doi:10.3389/fmolb.2022.1023131)
Supplement: Supplementary file 1 [file Table1.DOCX]

Supplementary Material

# Primers

**TABLE S1**. Utilized primers for SSM, amplification and sequencing of the FKBP51 coding sequence in pCT vector

| **Primer** | **Forward (fw) primer 5´-3´** |
| --- | --- |
| N63_deg_Fw | CATTACAAAGGAAAATTGTCANNKGGAAAGAAGTTTGATTCC |
| N63_deg_Rv | GACTGGAATCAAACTTCTTTCCMNNTGACAATTTTCC |
| G64_deg_Fw | CAAAGGAAAATTGTCAAATNNKAAGAAGTTTGATTCC |
| G64_deg_Rv | GACTGGAATCAAACTTCTTMNNATTTGACAATTTTCC |
| K65_deg_Fw | GGAAAATTGTCAAATGGANNKAAGTTTGATTCCAG |
| K65_deg_Rv | CTATCATGACTGGAATCAAACTTMNNTCCATTTGAC |
| K66_deg_Fw | GGAAAATTGTCAAATGGAAAGNNKTTTGATTCCAGTCATG |
| K66_deg_Rv | CTATCATGACTGGAATCAAAMNNCTTCCATTTGAC |
| F67_deg_Fw | GGAAAATTGTCAAATGGAAAGAAGNNKGATTCCAGTCATG |
| F67_deg_Rv | CATTTCTATCATGACTGGAATCMNNCTTCTTTCCATTTGAC |
| D68_deg_Fw | CAAATGGAAAGAAGTTTNNKTCCAGTCATGATAGAAATG |
| D68_deg_Rv | GGTTCATTTCTATCATGACTGGAMNNAAACTTCTTTCC |
| S69_deg_Fw | GTCAAATGGAAAGAAGTTTGATNNKAGTCATGATAG |
| S69_deg_Rv | GGTTCATTTCTATCATGACTMNNATCAAACTTCTTTCC |
| S70_deg_Fw | GGAAAGAAGTTTGATTCCNNKCATGATAGAAATG |
| S70_deg_Rv | GGTTCATTTCTATCATGMNNGGAATCAAACTTC |
| pCT_FKBP51_fw | AGTGGTGGTGGTGGTTCTGGTGGTGGTGGTTCTGGTGGTGGTGGTTCTGCTAGCATGAC |
| pCT_FKBP51_rv | TGTTGTTATCAGATCTCGAGCTATTACAAGTCCTCTTCAGAAATAAGCTTTTGCTCGGATCC |
| pCT_seq_up | TACCCATACGACGTTCCAGACTAC |
| pCT_seq_lo | CAGTGGGAACAAAGTCGATTTTGTTAC |

NNK is a degenerated codon with N = any nucleotide and K = G or C. MNN is the complementary codon with M = A or T.

# FACS analysis


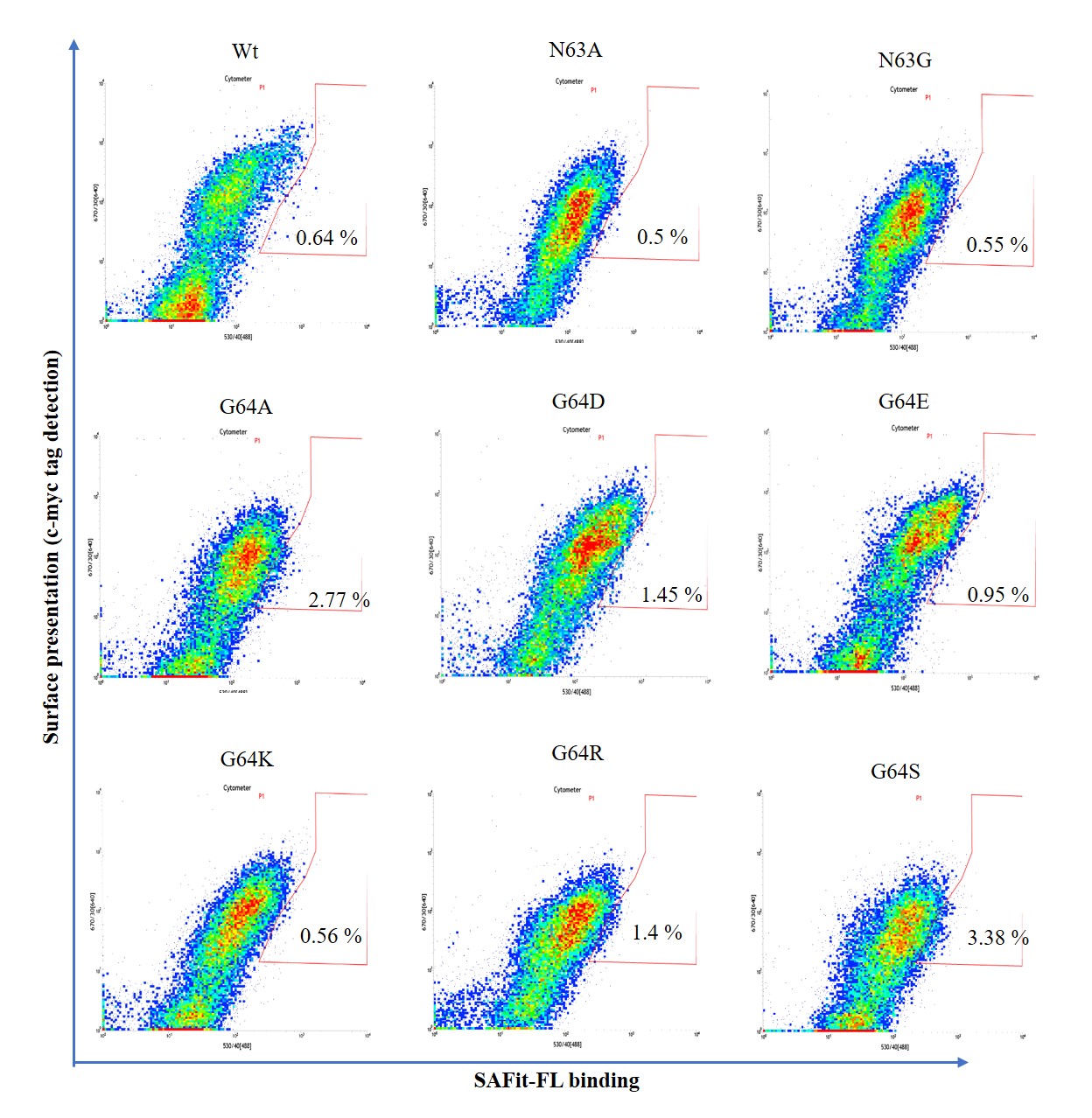


**FIGURE S1.** Individual dot-plots of the YSD presenting FKBP51 Wt and variants measured in a flow cytometer (part 1 of 3). Cells showing both surface presentation (c-myc tag detection) and SAFit‑FL_(out/in)_ binding signal are presented in the polygonal gate. Clones were sorted due to the general population shift close to the established gate


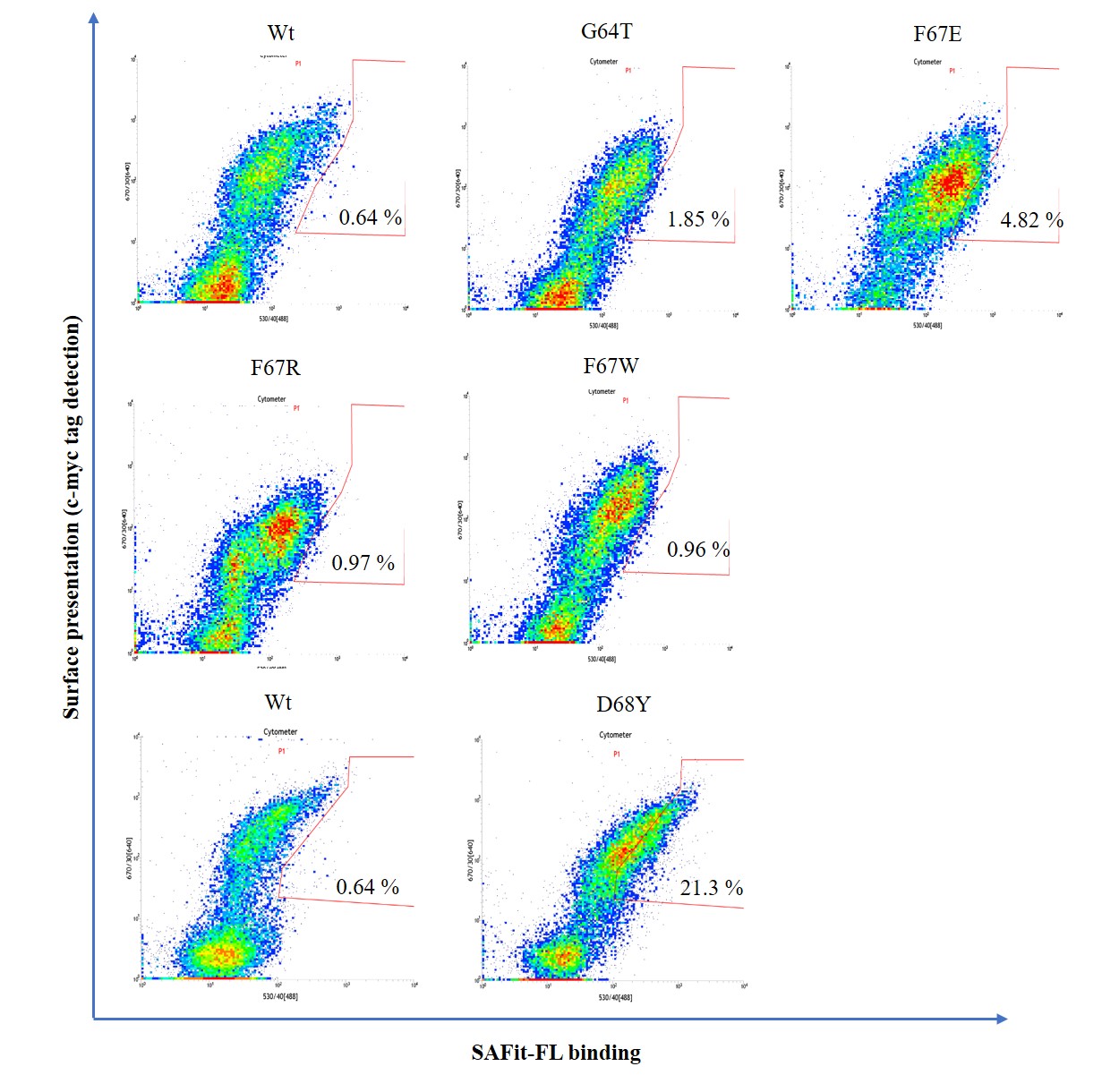


**FIGURE S2.** Individual dot-plots of the YSD presenting FKBP51 Wt and variants measured in a flow cytometer (part 2 of 3). Cells showing both surface presentation (c-myc tag detection) and SAFit‑FL_(out/in)_ binding signal are presented in the polygonal gate. Clones were sorted due to the general population shift close to the established gate


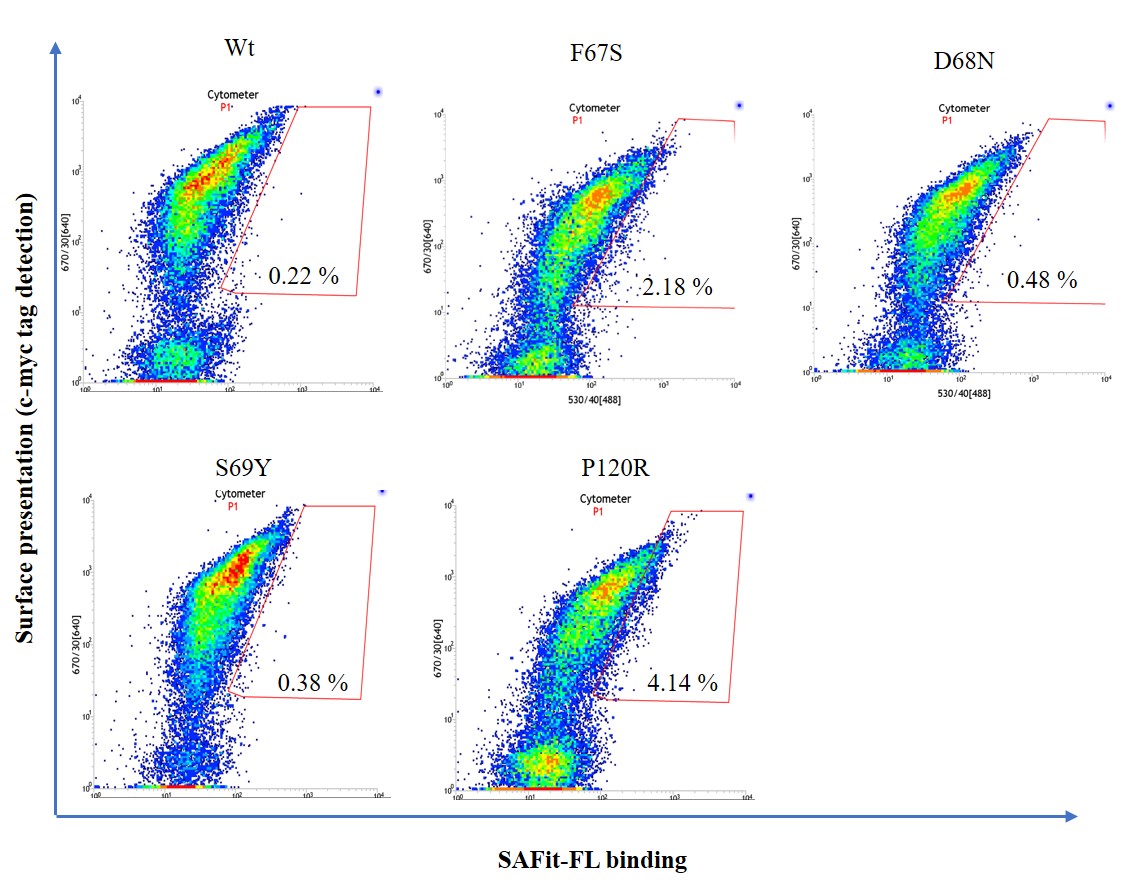


**FIGURE S3**. Individual dot-plots of the YSD presenting FKBP51 Wt and variants measured in a flow cytometer (part 3 of 3). Cells showing both surface presentation (c-myc tag detection) and SAFit‑FL_(out/in)_ binding signal are presented in the polygonal gate. Clones were sorted due to the general population shift close to the established gate


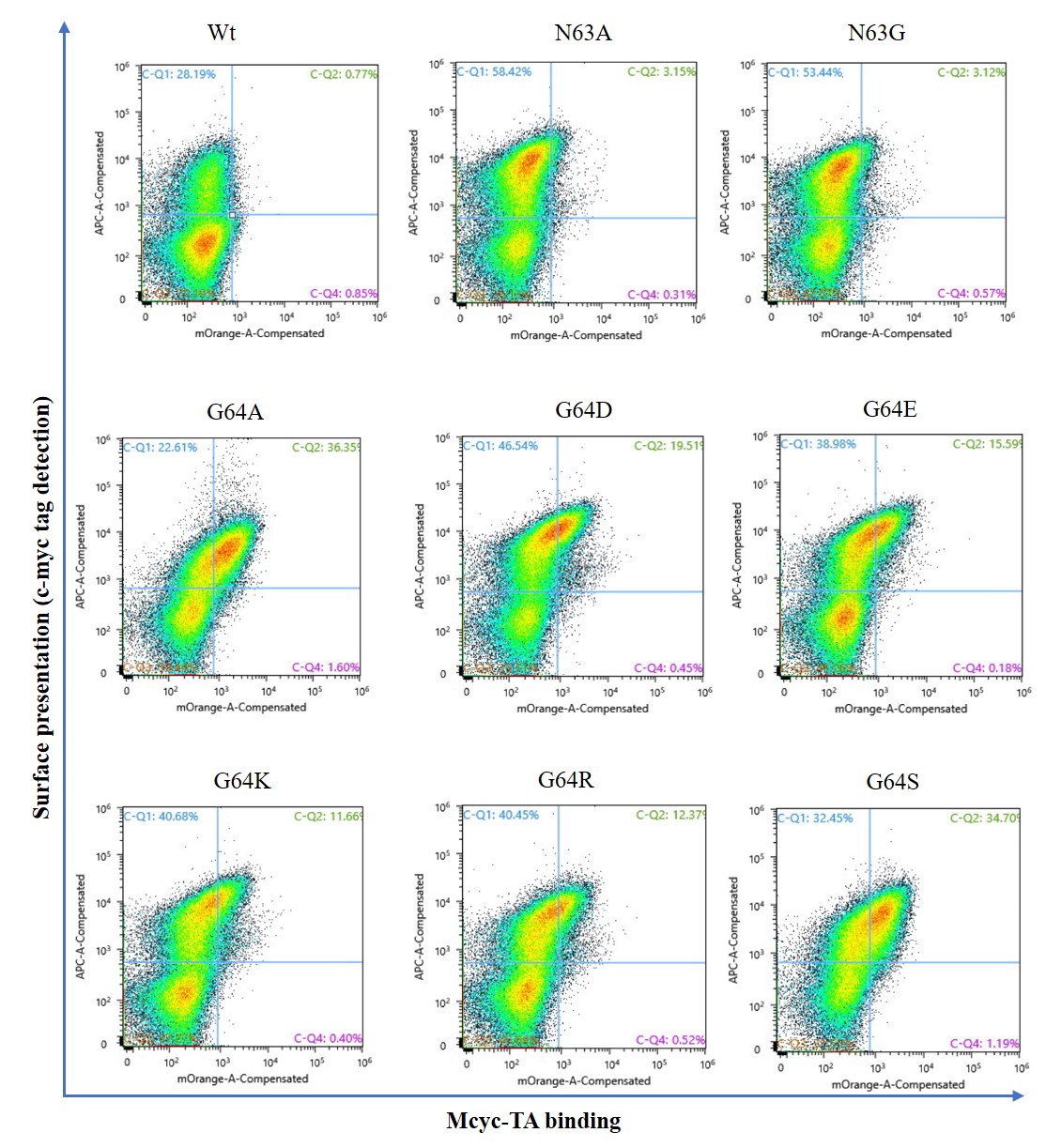


**FIGURE S4**. Individual dot-plots of the YSD presenting FKBP51 Wt and variants measured in a flow cytometer (part 1 of 2). Cells showing both surface presentation (c-myc tag detection) and Mcyc‑TA_(out/out)_ binding signal are presented in the second quadrant.


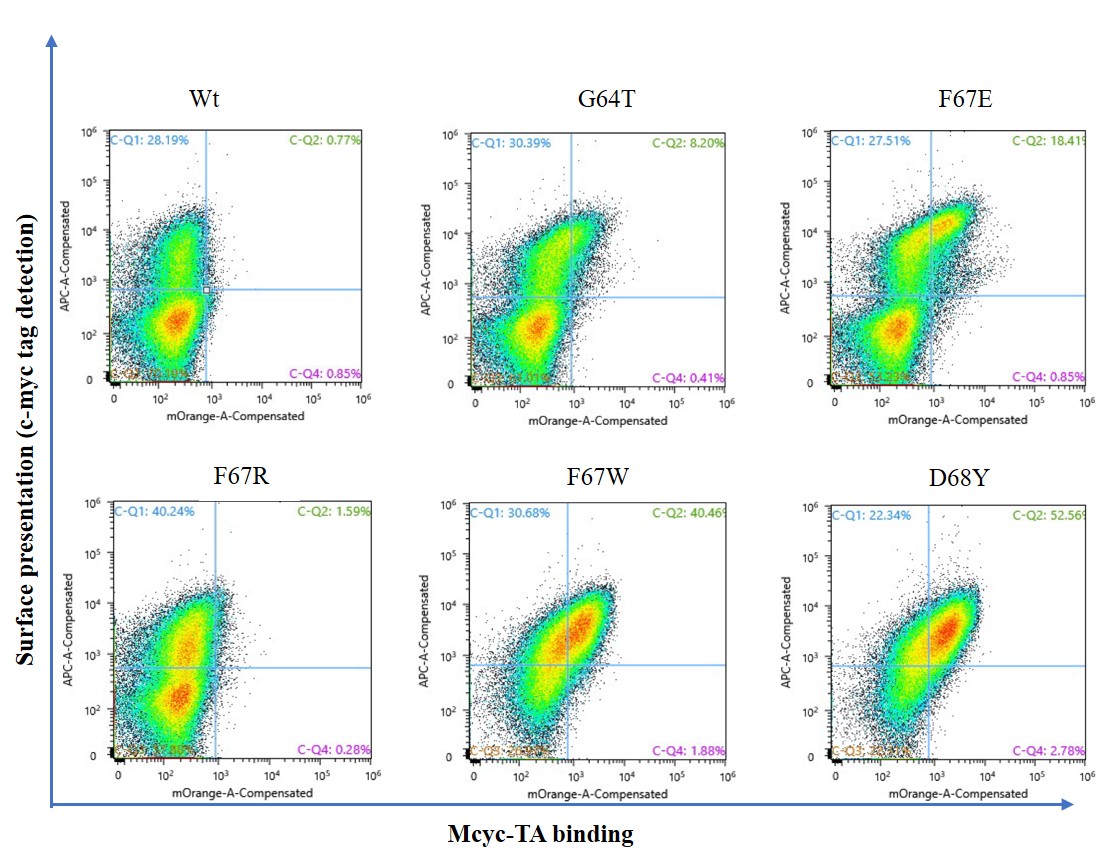


**FIGURE S5.** Individual dot-plots of the YSD presenting FKBP51 Wt and variants measured in a flow cytometer (part 2 of 2). Cells showing both surface presentation (c-myc tag detection) and Mcyc‑TA _(out/out)_ binding signal are presented in the second quadrant.

# Fluorescence polarization


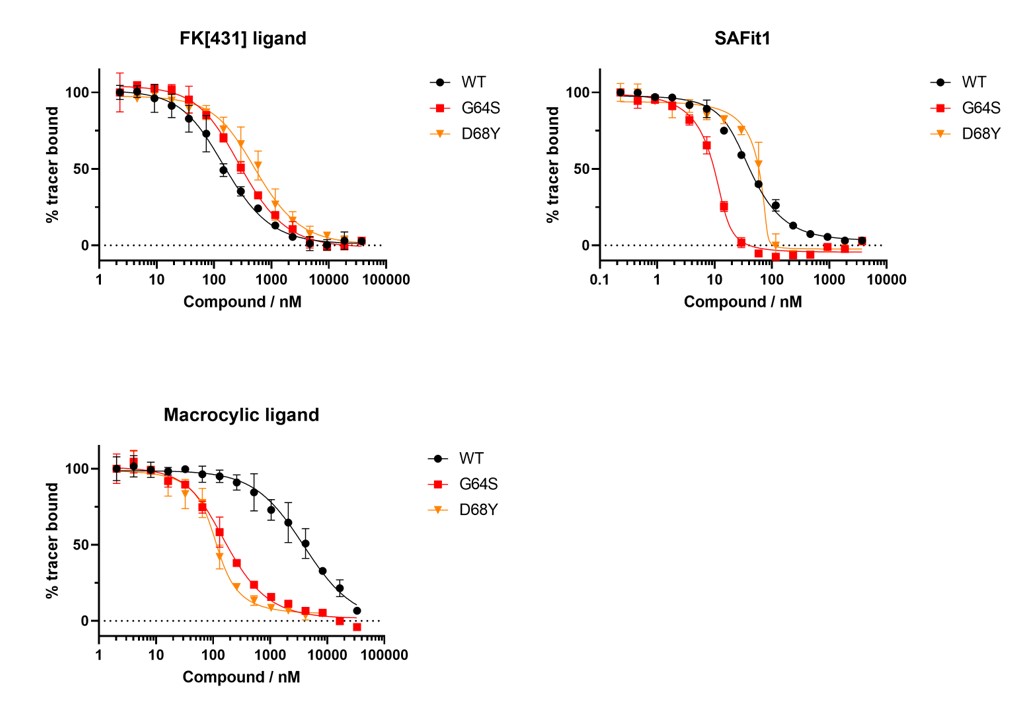


**FIGURE S6.** Competitive fluorescence polarization assays. FK[431] ligand _(in/in)_, SAFit1 _(out/in)_ or a macrocyclic ligand _(out/out)_ with FKBP51 WT, 10-40 nM G64S or 80-100 nM D68Y (without fluorophores) and 1 nM of the FK[431]-based tracer _(in/in)_ were used to rule out any effects of the fluorophores on the binding of the ligands to the FKBP51 variants.

**TABLE S2.** Ligand binding constants measured by competitive fluorescence polarization assays with the canonical FK[431] ligand _(in/in)_ and the two FKB51 specific ligands (SAFit1 _(out/in)_ and Mcyclic_(out/out)_)

| **FKBP51 variant** | **FK[431]-16h [Kd-value / nM]** | **SAFit1**  **[Kd-value / nM]** | **Macrocyclic ligand**  **[Kd-value / nM]** |
| --- | --- | --- | --- |
| WT | 27±3 | 4.8±0.4 | 780±100 |
| G64S | 56±4 | 0.5±0.2 | 29±3 |
| D68Y | 126±14 | 0.2±0.5 | 10±1 |

# Protein characterization


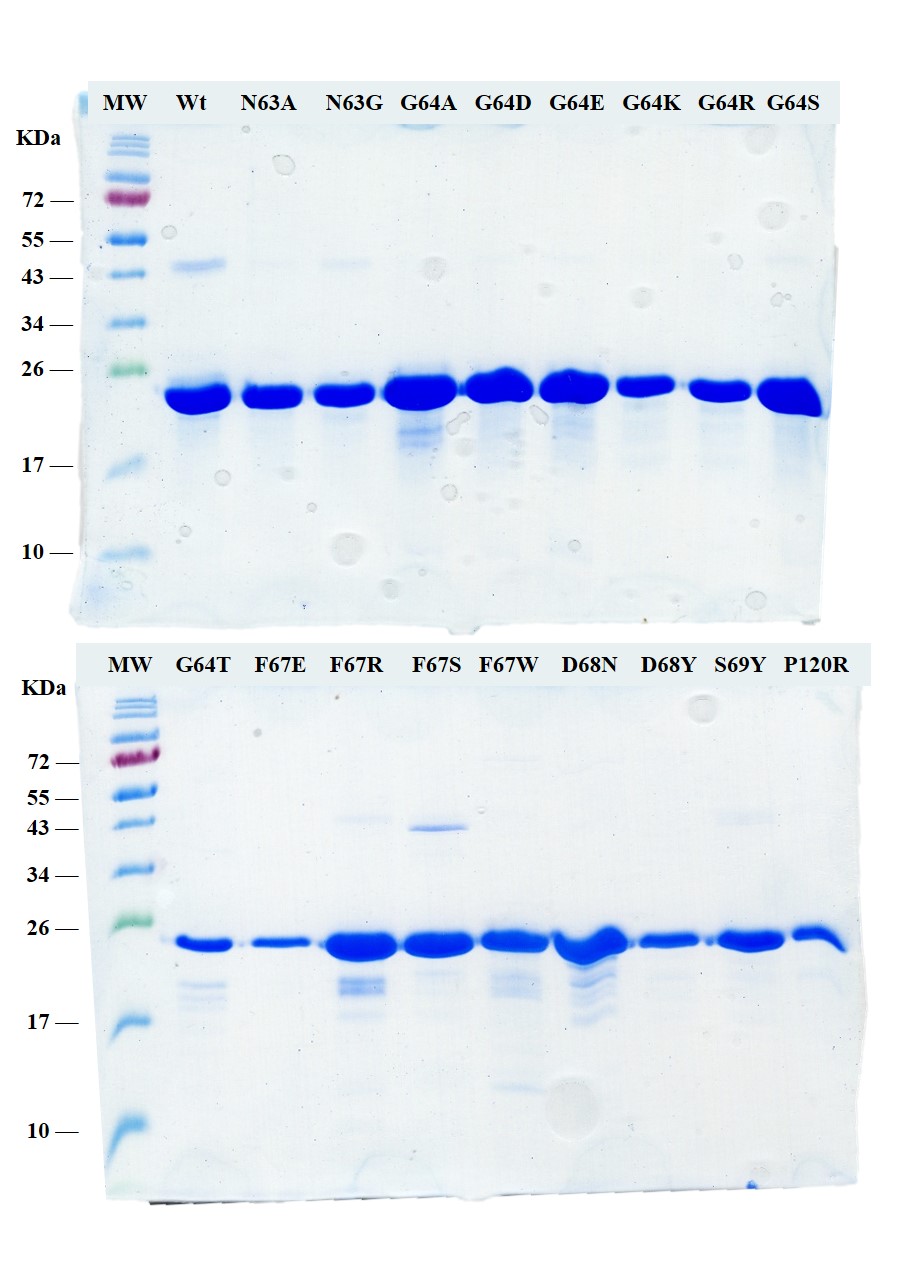


**FIGURE S7.** SDS-PAGE analysis of all FKBP51 variants after IMAC purification and dialysis in PBS. A molecular weight of ~19 KDa is expected for all the reduced samples.

# Tracers and ligands


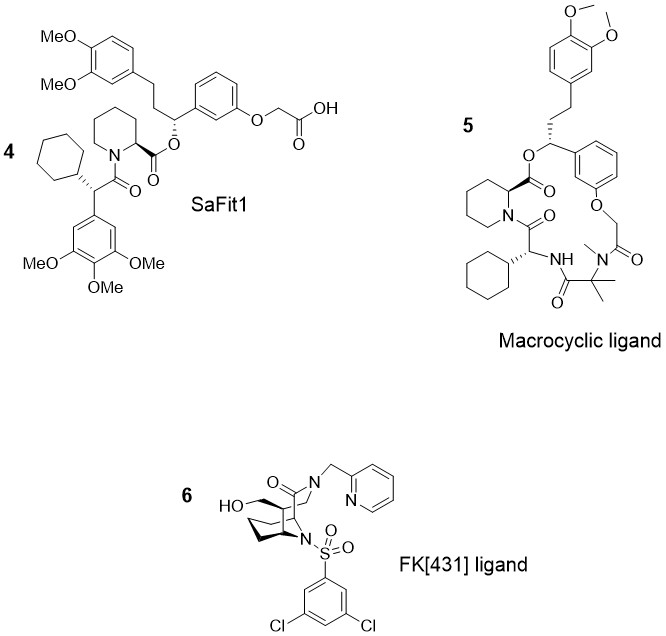


**FIGURE S8.** Chemical structure of the unlabeled FKBP51 ligands. The two FKBP51-spelective ligands SAFit1 (4) and macrocyclic ligand (5); these correspond to compounds SAFit1 from Gaali et al., Nat. Chem. Biol. 2015, 11 (1), 33–37 and compound 13 from Voll et al., Angew. Chem. Int. Ed. 2021, 60 (24), 13257–13263, respectively. canonical FKBP inhibitor FK[431]-16g correspond to compound 16g from Pomplun et al., J. Med. Chem. 2018, 61, 3660–3673

# Protein structures


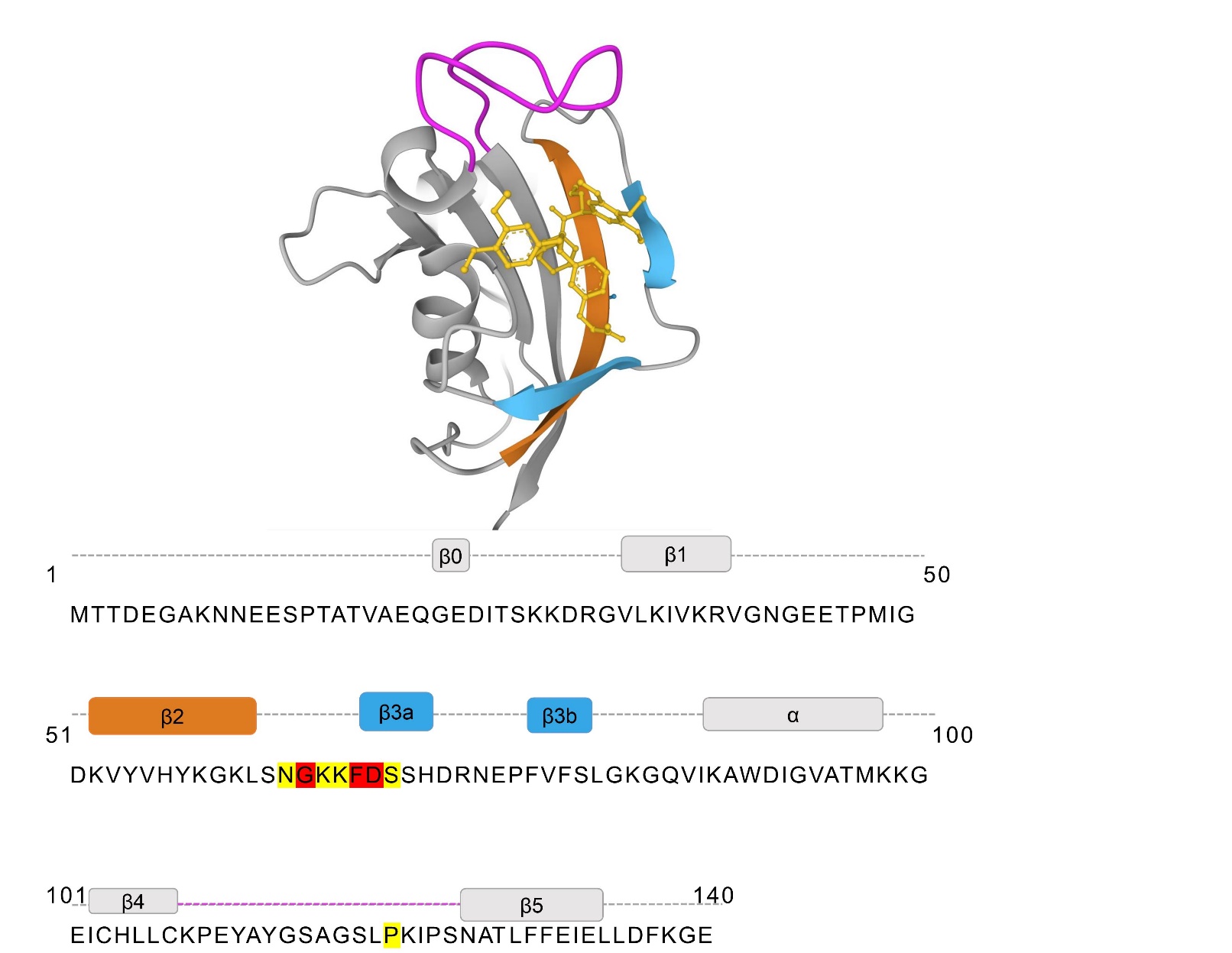


FIGURE S9. 3D structure of the FKBP51 FK1 domain (1-140) in complex with iFit1 (PDB: 4TW6).β2 and β3 strands are indicated in light blue, the β4-5 interconnecting loop is indicated in magenta and iFit1 is represented as golden sticks. The found variant positions of the protein-coding sequence are highlighted in yellow and the 3 best variants (G64, F67 and D68) positions identified by mutagenesis of the protein-coding sequence are highlighted in red.


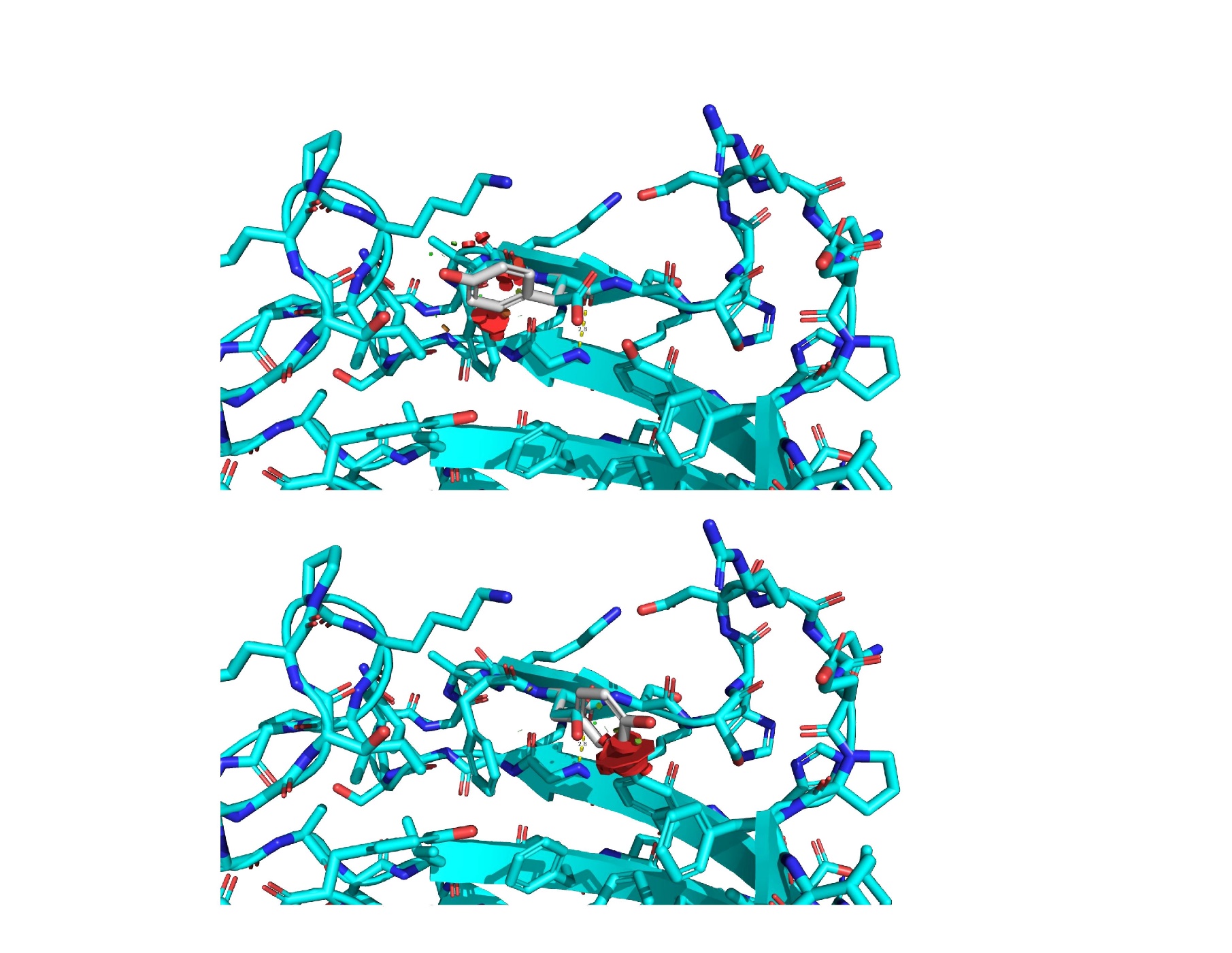


FIGURE S10. Modified structure of the FKBP51 FK1 domain in Apo state (amino acids: 14-140, PDB 3O5Q) with a D68Y amino acid exchange. The model demonstrates the steric clashes of the Y68 side chain in the canonical F67in/Y68in-conformation.


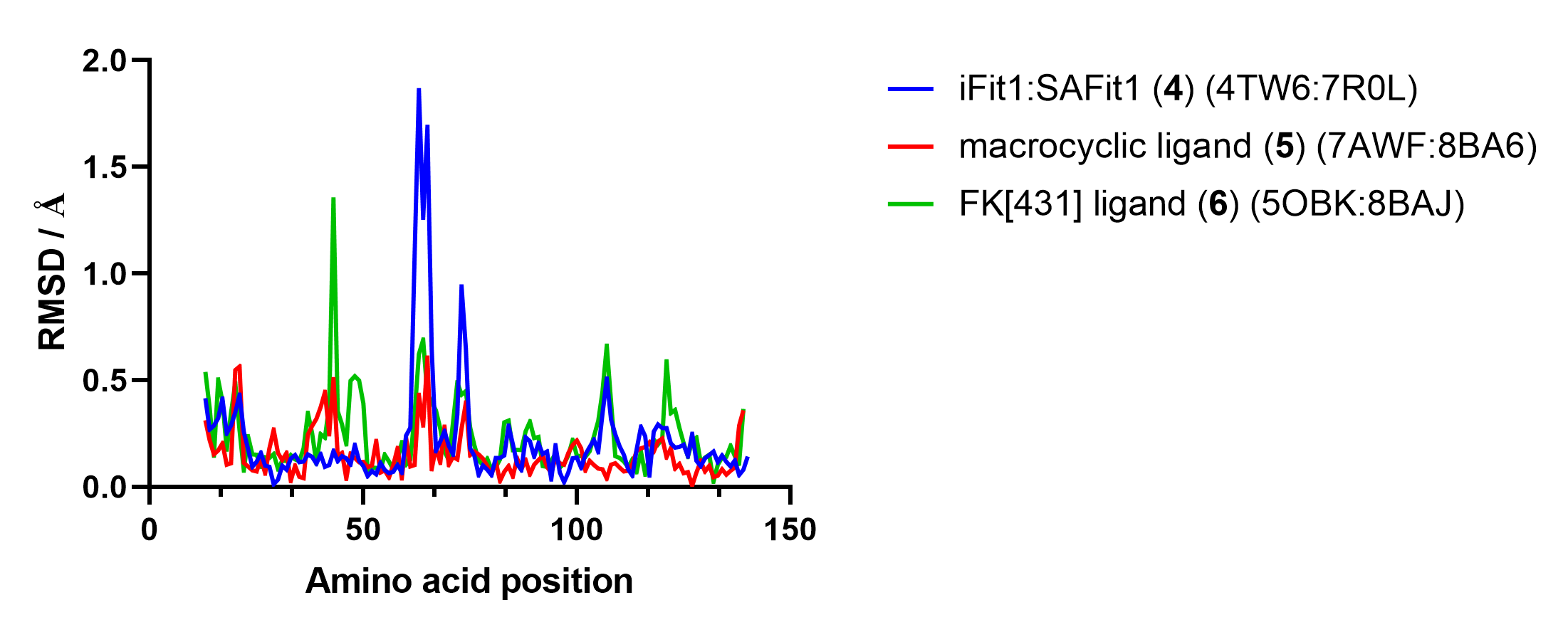


FIGURE S11. C_α_ RMSD values of a structural alignment of FKBP51-G64S in complex with SAFit1 (blue, PDB 7R0L), macrocyclic ligand (5) (red, PDB 8BA6) or FK[431] ligand (6) (green, PDB 8BAJ) aligned with structures of wild-type FKBP51 in complex with similar or the same ligands.

**TABLE S3.** φ/ψ angles of G64 of available FKBP51 apo structures or cocrystal structures with conformation-specific ligands.

| State | PDB-ID | φ of G64 | ψ of G64 |
| --- | --- | --- | --- |
| Apo (F67in/D68in) | 3O5E | 91.5° | -12.6° |
|  | 3O5G | 91.5° | -10.6° |
|  | 3O5Q | 91.9° | -9.5° |
| Canonical ligand bound (F67in/D68in) | 3O5R | 92.9° | -11.4° |
|  | 5OBK | 88.3° | -6.8° |
|  | 7APW | 89.3° | -8.8° |
| SAFit-like ligand bound (F67out/D68in) | 7A6X | 69.2° | 31.4° |
|  | 7B9Y | 67.9° | 25.2° |
|  | 7B9Z | 68.5° | 25.6° |
| Mcyc-like ligand bound (F67out/D68out) | 7AOT | -72.4° | 149.8° |
|  | 7AOU | -77.3° | 152.8° |
|  | 7AWF | -72.1° | 147.0° |

TABLE S4. Data collection and refinement statistics for the FK1 domain of the FKBP51 G64S variant structure of in complex with SAFit1 _(out/in)_.

| **PDB entry** | **7R0L** | **8BA6** | **8BAJ** |
| --- | --- | --- | --- |
| **Ligand** | SAFit1 | macrocyclic ligand **(5)** | FK[431] ligand **(6)** |
| **Data collection** |  |  |  |
| Beamline | BESSY II (BL14.1) | BESSY II (BL14.1) | BESSY II (BL14.1) |
| Wavelength | *λ* = 0.9184 Å | *λ* = 0.9184 Å | *λ* = 0.9184 Å |
| Space group | P2_1_2_1_2_1_ | P2_1_2_1_2_1_ | P2_1_2_1_2_1_ |
| Cell dimensions |  |  |  |
| *a, b, c* (Å) | 45.11, 48.59, 57.19 | 43.58, 50.42, 59.07 | 40.86, 54.24, 56.86 |
| *α, β, γ* (°) | 90, 90, 90 | 90, 90, 90 | 90, 90, 90 |
| Resolution (Å) | 37.00-1.10 (1.12-1.10) | 29.54-1.10 (1.12-1.10) | 39.28-1.20 (1.22-1.20) |
| *R*_merge_ | 0.067 (1.450) | 0.052 (0.781) | 0.115 (1.362) |
| *R*_pim_ | 0.039 (0.869) | 0.032 (0.348) | 0.068 (0.824) |
| *I/σ(I)* | 11.9 (1.3) | 16.5 (2.2) | 5.7 (1.2) |
| CC1/2 | 0.999 (0.633) | 1.000 (0.745) | 0.993 (0.559) |
| Completeness (%) | 99.9 (99.4) | 95.2 (89.7) | 98.4 (95.7) |
| Redundancy | 6.3 (6.1) | 6.6 (6.7) | 6.8 (6.8) |
| **Refinement** |  |  |  |
| Resolution (Å) | 37.00-1.10 | 29.55-1.10 | 39.28-1.-20 |
| No. of reflections | 51662 | 50756 | 39464 |
| *R*_work_/*R*_free_ (%) | 14.2/17.1 | 14.1/16.2 | 17.6/21.0 |
| No. of atoms |  |  |  |
| Protein | 1929 | 2060 | 1864 |
| Ligand | 106 | 100 | 53 |
| Water | 147 | 222 | 156 |
| *B*-factors |  |  |  |
| Protein | 15.9 | 13.3 | 15.1 |
| Ligand | 13.8 | 9.7 | 15.5 |
| Water | 34.7 | 30.7 | 29.4 |
| R.m.s. deviations |  |  |  |
| Bond lengths (Å) | 0.0192 | 0.0129 | 0.0108 |
| Bond angles (°) | 2.149 | 1.770 | 1.713 |
| Ramachandran plot |  |  |  |
| Favoured (%) | 97.00 | 98.00 | 97.00 |
| Allowed (%) | 3.00 | 2.00 | 3.00 |
| Outlier (%) | 0.00 | 0.00 | 0.00 |
